# *Corallocarpus glomeruliflorus*: Pharmacological potential revealed by phytochemical and in silico investigations

**DOI:** 10.1016/j.bbrep.2025.101940

**Published:** 2025-02-07

**Authors:** Fatima Saleh Naji Bin-Asal, Adel A.M. Saeed, Abdul-Rahman Alawi Bin Yahia

**Affiliations:** a Department of Chemistry, Faculty of Education, University of Aden, Aden, Yemen; b Department of Chemistry, Faculty of Science, University of Aden, Aden, Yemen; c Department of Pharmaceutical Chemistry, Faculty of Pharmacy, University of Aden, Yemen

**Keywords:** *Corallocarpus glomeruliflorus*, Methanolic extract, Bioactive compounds, Docking, Qualitative and quantitative analysis, Yemeni plant

## Abstract

*Corallocarpus glomeruliflorus* (CGP), a plant native to Yemen, has been traditionally used for the management of various health conditions, including cancer, inflammation, and diabetes. This study presents the first comprehensive phytochemical and pharmacological investigation of CGP, revealing novel molecular mechanisms and therapeutic potential. Phytochemical analysis of the CGP extract revealed the presence of diverse bioactive compounds, including phenols, flavonoids, and other secondary metabolites. Notably, this is the first report identifying maritimetin, assafoetidin, kaempferol 3-rhamnoside 7-xyloside, and lespenefril in CGP, compounds with significant therapeutic potential. The total phenolic content was 88.12 ± 4.48 mg GAE/g, significantly higher than previously reported for related species (63.78 ± 1.27 mg GAE/g), and the total flavonoid content was 22.1 ± 0.01 mg QE/g. The extract demonstrated superior antimicrobial activity against Pseudomonas aeruginosa compared to previous studies, with a zone of inhibition of 16.7 ± 1.53 mm at 200 mg/mL concentration. The CGP extract displayed strong antioxidant activity in DPPH, FRAP, and phosphomolybdenum assays, with an IC50 value of 48.39 ± 1.58 μg/mL in the DPPH assay, compared to 9.88 ± 0.54 μg/mL for the positive control ascorbic acid. Most significantly, the CGP extract exhibited more potent selective anticancer effects on human breast (MCF-7) and colon (HCT-116) cancer cell lines than previously reported for related Cucurbitaceae species, with IC50 values of 49.18 ± 2.81 μg/mL and 244.2 ± 9.86 μg/mL, respectively. Our novel molecular docking studies revealed previously unreported interactions between CGP compounds and key therapeutic targets, particularly Pim-1, PIK3CA, α-amylase, and Gyr-B, providing new insights into its mechanism of action.

## Highlights

• Comprehensive phytochemical analysis of *Corallocarpus glomeruliflorus* (CGP) extract using LC-MS.
• Potent antimicrobial, antioxidant, anticancer, anti-inflammatory, and antidiabetic activities of CGP.
• Selective cytotoxicity of CGP against breast (MCF-7) and colon (HCT-116) cancer cell lines.
• Significant inhibition of COX-2, 15-LOX, and α-amylase enzymes by CGP extract.
• In silico docking suggests Maritimetin, Kaempferol, and Lespenefril as key bioactive compounds.

## Graphical abstract

**Figure:**
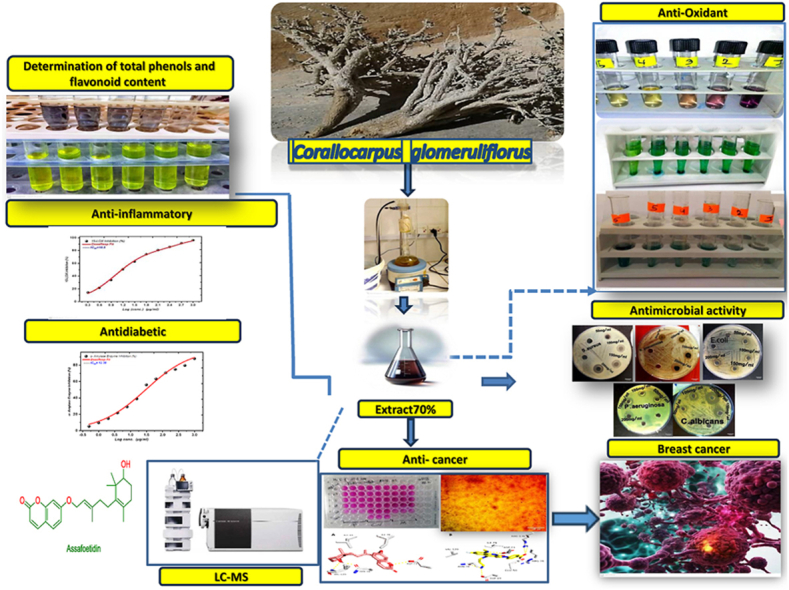

## 1 Introduction

Natural products have gained increasing attention as potential sources for the development of novel medicines, nutraceuticals, and cosmetics [[1], [2], [3]]. The *Cucurbitaceae* family, widely distributed globally, is known for its long history of traditional medicinal uses and a wide range of pharmacological activities [4].

Cucurbitaceae plants are often characterized by the presence of unique secondary metabolites, such as triterpenoids, sterols, and cucurbitacins, which contribute to their diverse therapeutic effects [5]. Some members of this family have been reported to possess anti-inflammatory, antioxidant, antimicrobial, anti-diabetic, and anti-cancer properties, making them a valuable source for drug discovery and development [4,5].

*Corallocarpus glomeruliflorus*, commonly known as yellow roots, is a member of the *Cucurbitaceae* family and is renowned for its exceptional medicinal properties. The plant is indigenous to Yemen and Oman and contains high concentrations of phytochemicals, including steroids, terpenoids, glycosides, alkaloids, saponins, and tannins. These bioactive compounds have been linked to a range of therapeutic effects, such as anti-inflammatory, anti-aging, phlebotonic, spasmolytic, diuretic, and sedative activities [6,7].

Scientific studies have validated the traditional use of *C. glomeruliflorus*, emphasizing its analgesic, hypolipidemic, antihyperglycemic, anticancer, antibacterial, anti-inflammatory, antistress, and immunomodulatory properties [7].

Cancer, particularly breast cancer and colorectal cancer, continues to be a serious global health concern with significant impacts on human health [[8], [9], [10]]. Over 50 % of anticancer drugs have plant-based origins, underscoring the significant role of medicinal plants in oncology [11]. *Corallocarpus glomeruliflorus*, with its rich phytochemical composition, represents a promising candidate for further research into novel anticancer agents that could potentially mitigate the severe side effects of conventional chemotherapy.

Diabetes mellitus, a metabolic condition characterized by chronic hyperglycemia, is another major health concern worldwide [12,13]. One effective therapeutic approach for managing postprandial hyperglycemia involves inhibiting carbohydrate-hydrolyzing enzymes such as glucosidase and amylase, thereby delaying glucose absorption [14]. While synthetic inhibitors like acarbose are available, their cost limits widespread use in developing nations. In contrast, naturally occurring compounds in plants have shown promise in managing postprandial hyperglycemia effectively and affordably [15].

Traditional herbal medicines, including *C. glomeruliflorus*, have been used extensively for diabetes and cancer management [11,16,17]. Exploring the medicinal properties of *C. glomeruliflorus* is crucial, as the plant's diverse phytochemical composition offers a wealth of therapeutic potential, particularly in the treatment of diabetes and cancer.

By delving into the unique properties of plant-derived active compounds, scientists can uncover mechanisms of action that may lead to the formulation of effective therapeutics. This exploration not only advances our understanding of plant biology but also opens up new avenues for pharmaceutical applications.

## 2 Material and methods

### 2.1 Collection and preparation of plant material

Stems and twigs aerial components of *C. glomeruliflorus* plant were harvested from Yemen's Hadramout Governorate during October 2021. The plant was identified and authenticated by a taxonomist professor Othman Saad Saeed Al-Hawshabi at the Botany Department, Faculty of Science, University of Aden, Yemen. A voucher specimen (No. CG-2022-001) was deposited in the herbarium of the same department for future reference.

Post-collection processing involved sequential washing with regular water followed by double-distilled water for thorough cleaning. The plant materials were cut into smaller segments and allowed to dry naturally in shaded conditions at ambient temperature. A mechanical grinder was employed to process the dried material into a uniform powder, which was then stored in airtight containers until further analysis. The extraction process utilized 50 g of powdered material combined with 500 mL methanol (70 %) in a Soxhlet apparatus to obtain the crude methanolic extract, which was subsequently concentrated, removed the solvent and preserved at −20 °C for analytical procedures.

### 2.2 Physico-chemical analysis and phytochemical profiling

Physicochemical parameters were assessed following the standard quality control methods for medicinal plant materials. The crude extracts underwent preliminary phytochemical screening of the CGP methanolic extract was performed to detect the presence of various secondary metabolites, including alkaloids, flavonoids, terpenoids, steroids, tannins, and saponins, using standard protocols [[18], [19], [20]].

#### 2.2.1 Qualitative analysis

The intensity of the color reactions was illustrated by symbols: (−) for no observed changes and therefore negative results, (+) for low-intensity positive results, (++) for mild intensity positive results, and (+++) for very high-intensity positive results.

The mass spectrometry investigations were carried out using Agilent 6550 QTOF-MS/MS equipment in conjunction with an Agilent 1200 HPLC (Agilent Technologies, USA). The complete scan of mass spectra were attained at a scan rate of 1.00 and within the range of *m*/*z*, amu 120–1200. The first time the valve was switched on, it was with an injection volume of 5.00 μl [21,22].

#### 2.2.2 Quantitative analysis

##### 2.2.2.1 Determination of total phenols content (TPC)

To quantify the total phenols content (TPC), the Folin-Ciocalteu method was applied. This involved mixing a 0.5 mL of the sample extract, 2.5 mL of 10 % (v/v) Folin-Ciocalteu and 2 mL 7.5 % (w/v) sodium carbonate solution, respectively. After allowing the resulting mixture to react for one and half houres, its absorbance was measured at 765 nm. Various gallic acid solutions, with concentrations between 55 and 95 μg/mL, were utilized in establishing a calibration curve. The total phenolic content (TPC) was subsequently quantified in milligrams of gallic acid equivalents (GAE) per gram of extract [23].

##### 2.2.2.2 Determination of total flavonoid content (TFC)

The process described in Ref. [23] was followed in order to evaluate the extracts' total flavonoid content using the aluminum chloride colorimetric technique. 0.5 mL of extract, 1.5 mL of methanol, 0.1 mL of 1 M potassium acetate solution, 2.8 mL of **double-**distilled water, and 0.1 mL of 10 % AlCl_3_ solution were combined in the procedure. After that, the mixes were allowed to incubate for half an hour at room temperature. A UV–vis spectral technique was then accomplished to test the solutions' absorbance at 430 nm. A series of quercetin concentrations, ranging from 20 to 40 μg/mL, was employed to develop the typical curve. The data were denoted as milligrams of Quercetin Equivalents (QE) per gram of extract.

### 2.3 Preparation of microbial suspension

Bacterial strains, including *Escherichia coli*, *Pseudomonas aeruginosa*, *Staphylococcus aureus*, and *Klebsiella pneumoniae*, were sourced from Médecins Sans Frontières (MSF). Additionally, the Candida albicans fungus was isolated from vaginal samples and subsequently diagnosed. For the preparation of the stock solution, 1 g of the crude extract was dissolved in 5 mL of DMSO, resulting in a concentration of 200 mg/mL. A suspension of bacteria or fungi was created by transferring several colonies, no older than 24 h, into a test tube containing sterile 0.9 % saline solution. The turbidity of this suspension was adjusted to align with the 0.5 McFarland standard, which is approximately 1–2 × 10^8^ CFU/mL for bacteria and 1–5 × 10^6^ CFU/mL for fungi. To assess the antibacterial and antifungal activities of the plant extract, a modified agar well diffusion method was utilized. Using a sterile cotton swab, each bacterial strain was uniformly distributed on Mueller-Hinton Agar (MHA) plates. Then, using a sterile cork borer, wells of 6 mm in diameter were made in the agar. Then, 50 μL of the plant extract was applied to each well at different quantities (50, 100, 150, and 200 mg/mL). Within 18–24 h of the plates being incubated at 36 °C, the zones of growth inhibition surrounding the wells were assessed [24].

### 2.4 Antioxidant activity

The antioxidant capacity evaluation employed multiple complementary assays. The DPPH radical scavenging assessment utilized a freshly prepared 1 mM DPPH solution in methanol. The extract was tested across concentrations ranging from 31.25 to 1000 μg/mL, with ascorbic acid serving as the reference standard. Following 30-min dark incubation, measurements were recorded at 517 nm using spectrophotometric analysis. The ferric reducing antioxidant power (FRAP) determination involved combining the extract with phosphate buffer and potassium ferricyanide, followed by trichloroacetic acid addition and ferric chloride reaction. The phosphomolybdenum assay evaluated total antioxidant capacity through the reduction of Mo(VI) to Mo(V), measuring the resulting green phosphomolybdenum complex spectrophotometrically at 695 nm [[25], [26], [27]].

### 2.5 Cell culture and cytotoxicity

Human cancer cell lines MCF-7 (breast adenocarcinoma) and HCT-116 (colorectal cancer) were maintained in appropriate growth media supplemented with 10 % heat-inactivated fetal bovine serum and antibiotics under standard culture conditions (37 °C, 5 % CO_2_). The cytotoxicity assessment employed the SRB assay protocol. Cells were seeded in 96-well plates (5 × 10^3^ cells/well) and exposed to varying extract concentrations for 72 h. Post-treatment cells underwent fixation with TCA, staining with SRB solution, and subsequent solubilization with Tris buffer for absorbance measurement at 540 nm [28,29].

### 2.6 Enzyme inhibition studies

#### 2.6.1 Human cyclooxygenase (COX) inhibition assay

As a sensitive spectrophotometric assay for prostaglandin synthase activity, the hydroperoxide generated in the cyclooxygenase reaction can oxidize 1-dichlorofluorescein (1-DCF) in the presence of phenol.

A solution of 1 M NaOH (50 μL) was used to hydrolyze 5 mg of leuco-2,7-dichlorofluorescein diacetate at room temperature for 10 min. To remove any surplus NaOH, 30 μL of 1 M HCl was added. Diluted in 0.1 M Tris buffer, pH8, the resultant leuco-dichloro fluorescein (1-DCF) was carefully handled. A solution of 0.1 M Tris buffer, pH8, was used to dilute the cyclooxygenase enzyme (COX-2). The enzyme was pre-incubated with the test extracts or standard (20 μL) at room temperature for 5 min with hematin present. The enzyme mixture was supplemented with pre-mixed phenol, 1-DCF, and arachidonic acid to initiate the reaction. The final reaction mixture consisted of 50 μM arachidonic acid, 500 μM phenol, 20 μM 1-DCF, and 1 μM hematin in a 1 mL final volume of 0.1 M Tris-buffer, pH8.

A UV–visible spectrophotometer (Milton Roy, Spectronic 1201) was used to record the reaction spectrophotometrically for 1 min at 502 nm [30,31]. If the test sample was found to have any non-enzymatic activity, it was corrected by comparing each test reaction to a blank reaction mixture in the spectrophotometer reference cell. The reaction mixture without the enzyme was called the blank. The compound celecoxib was utilized as the standard.

The percent inhibition for each inhibitor can be calculated using the following equation:

(1) $$\text{Inhibition}\;\left(\%\right)=\frac{\mathrm{Activity}\;\mathrm{of}\;\mathrm{Control}-\mathrm{Activity}\;\mathrm{of}\;\mathrm{Test}}{\mathrm{Activity}\;\mathrm{of}\;\mathrm{Control}\;}\times 100$$

#### 2.6.2 Lipoxygenase (LOX) inhibition assay

The human recombinant enzyme assay kit (catalog no 760700, Cayman Chemical, Ann Arbor, MI, USA) was used by the manufacturer's instructions to ascertain the compounds' capacity to inhibit 15-LOX enzymes (IC50 values, μg/mL). Shortly before usage, the stock and buffer solutions (0.1 M Tris HCl, pH7.4) were prepared. 10 μL of each extract was prepared, dissolved in the smallest quantity of DMSO, and then diluted with the stock solution in order to reach concentrations of (0.5, 1, 2, 3.9, 7.8, 15.6, 31.25, 62.5, 125, 250, 500, and 1000 μg/mL) in a final volume of 210 μL. The 15-LOX was determined by measuring the increase in absorbance at 490 nm using a microplate reader (Biotek, USA) [32].

The inhibition levels of the compounds were determined using Equation (1). Each sample, along with blanks and standards, was tested in triplicate. NDGA was employed as a positive control for this study.

The IC50 value, representing the concentration of the test compound that resulted in 50 % inhibition, was determined by analyzing the concentration-response curve through Origin software.

### 2.7 α-Amylase inhibitor assay

140 μL of phosphate buffer (50 mM, 0.9 % NaCl, pH7) was mixed with 20 μL of samples or blanks in 96-microwell plates. The mixture was then incubated for 15 min at 37 °C after 20 μL of amylase enzyme (1 mg/mL in the same buffer) was added. After this incubation, 20 μL of substrate was added to the mixture at a final concentration of 0.375 mM in phosphate buffer, and it was then incubated again for 10 min at 37 °C. A microplate reader (Onega, USA) was used to measure the release of p-nitroaniline from the substrate at a wavelength of 405 nm in order to determine the enzyme activity [33]. The following equation was used to determine the percentage of α-amylase inhibition:

(2) $$\text{Inhibition}\;\left(\%\right)=\frac{A_{\mathrm{Blank}}–\;A_{\mathrm{Sample}}}{A_{\mathrm{Blank}}}\times 100$$

Where, A_Blank_ is the absorbance of the control (blank, without inhibitor), and A_Sample_ is the absorbance in the presence of the inhibitor.

### 2.8 Statistical analysis

Statistical analyses were conducted using Origin 9 software, implementing a comprehensive approach to data evaluation. Descriptive statistics included mean values ± standard deviation (SD). Comparative analyses employed one-way ANOVA for multiple group comparisons, supplemented by Student's t-test for paired comparisons and Tukey's HSD post-hoc tests where appropriate. Significance criteria were established at p<0.05, with exact p-values reported when possible, accompanied by effect size calculations. The validation metrics incorporated Chi-square (χ2) analysis for categorical data, F-values for ANOVA results, and R^2^ values for correlation analyses. All experiments were performed in triplicate (N = 3) with appropriate technical replicates.

Phenolic and flavonoid content variations were analyzed through one-way ANOVA. Activity comparisons between extract and reference compounds underwent Student's t-test analysis, with significance established at p<0.05. Chi-square (χ^2^) analysis provided additional statistical validation for observed effects. For antioxidant, anticancer, anti-inflammatory, and antidiabetic activities, both t-values and p-values were calculated to ensure robust statistical interpretation.

## 3 Results and discussion

The results of this study provide critical insights into the phytochemical composition and diverse biological activities of CGP, highlighting its potential therapeutic applications.

### 3.1 Physicochemical profiling of CGP

The physicochemical properties of the *C. glomeruliflorus* powder were analyzed, and the results are summarized in Table 1. The loss on drying at 137 °C was found to be 9.2 %, indicating the moisture content of the sample. The total ash content, which reflects the total amount of inorganic material, was measured at 19.87 %. The acid-insoluble ash content, representing the non-dissolvable inorganic residue in acid, was 0.7 %. Water-soluble ash, which indicates the portion of ash content soluble in water, was 13.32 %. The pHoftheextractwasdeterminedtobe7.5, suggesting a neutral pH. Lastly, the extractive value, which assesses the amount of active constituents extractable from the plant material, was recorded at 17 % indicating the abundance of bioactive phytochemicals that could be successfully recovered.

**Table 1:** Physicochemical analysis of CGP.

No.	Parameter	Value%
1	Loss on Drying at 137° C	9.2 %
2	Total Ash (%)	19.87 %
3	Acid in Soluble Ash(%)	0.7 %
4	Water Soluble Ash(%)	13.32 %
5	PH	7.5 %

### 3.2 Qualitative and quantitative analysis

The methanolic extract of *Corallocarpus glomeruliflorus* (CGP) was found to contain a diverse array of phytochemicals, as revealed by the qualitative and quantitative analyses.

#### 3.2.1 Qualitative analysis

Alkaloids, anthraquinones, flavonoids, glycosides, saponins, terpenoids, steroids, carbohydrates, tannins, phenols, proteins, and coumarins were all found in the CGP extract during the initial phytochemical screening (Table 2). The intensity of the color reactions indicated the relative abundance of these secondary metabolites.

**Table 2:** Qualitative analysis of CGP.

Constituent	Reagent test	Positive result	Observation	Remark
Alkaloids	Mayer's	Creamy precipitate	++	Moderately present
	Wagner's	Reddish-brown precipitate	+++	Abundantly present
	Dragendroff's	Red precipitate	+++	Abundantly present
Anthraquinones	Modified Borntrager's test	Pink/red or violet color in the lower	+++	Abundantly present
Flavonoids	Shinoda test	Pink scarlet	+++	Abundantly present
	C_2_H_5_OH/KOH	Yellow color	+++	Abundantly present
Glycosides	Keller- Kiliani test	Reddish brown coloration	+++	Abundantly present
Saponins	Foam test	Foam for 10min	+++	Abundantly present
Terpenoids	Salkowski test	Reddish brown coloration	+++	Abundantly present
Steroids	Lieberman – burchard ‘s test	Greenish color	++	Moderately present
Carbohydrates	Fehling's test	A reddish precipitate	+++	Abundantly present
	Molisch's	Reddish violet color ring	+++	Abundantly present
	Benedict's test	A reddish precipitate	+++	Abundantly present
Tannins	Ferric chloride (FeCl_3_)	Blackish blue color	–	Negative test
	lead acetate	Yellow/white precipitate	+++	Abundantly present
Phenols	ethanol + pinch of FeCl_3_	Greenish yellow color	+++	Abundantly present
	1 % (FeCl_3_ potassium ferrocyanide	A greenish-blue color	+++	Abundantly present
Proteins	Biuret test	Violet color	+	Slightly present
Phlobatinins	1 % HCl	Red precipitate	–	Negative test
Quinones	Conc. HCl	Green color	–	Negative test
Coumarins	10 % NaOH	Yellow color	+++	Abundantly present

Legend: + = Slightly present, ++ = Moderately present, +++ = Abundantly present, - = Not found.

The phytochemical profiling of the CGP extract revealed the presence of various classes of bioactive compounds, including flavonoids, terpenoids, and alkaloids. These findings align with previous reports on similar species and underscore the potential anticancer properties of these compounds [[18], [19], [20]].

#### 3.2.2 LC-MS analysis

The LC-MS analyses offered additional understanding of the phytochemical of the CGP extract (see Supplementary materials-Table S1). The metabolite profile of the methanolic extract from *Corallocarpus glomeruliflorus* was assessed utilizing liquid chromatography-mass spectrometry (LC-MS) methodologies [25,26]. To obtain a thorough characterization of the compounds within the extract, both negative and positive ionization modes were utilized.

In the positive ionization mode, several compounds were identified. Several derivatives of kaempferol, such as Kaempferol3-rhamnoside7-xyloside, were detected. Kaempferol itself is recognized for its anti-inflammatory, anti-cancer, and antioxidant effects. Assafoetidin which have been shown to exhibit anti-cancer [34], In the negative ionization mode, phenolic compounds and other including maritimetin, and Lespenefril were detected. These phenolic substances are recognized for their properties, which include anti-inflammatory, anti-diabetic, antioxidant, and anti-cancer effects [35,36].

The presence of these diverse bioactive compounds, as revealed by the phytochemical analysis, provides a plausible explanation for the wide range of pharmacological activities exhibited by the CGP extract, including its antimicrobial, antioxidant, anti-inflammatory, anti-cancer, and anti-diabetic effects.

The varied phytochemical profile of the CGPextract, as indicated by qualitative and LC-MS analyses, supports its traditional use in the treatment of numerous health conditions, including cancer, inflammation, and diabetes. The interactions among these bioactive elements likely enhance the broad spectrum of pharmacological effects associated with the CGP extract.

### 3.3 Quantitative analysis

#### 3.3.1 Determination of total phenols content (TPC) and total flavonoid content (TFC)

The total phenolic and flavonoid content of the extracts are shown as mg GAEs/g of the CGP and mg QEs/g of the extract, respectively, as shown in Figs. S1 and S2. The total phenolic content (88.12 ± 4.48 mg GAE/g extract; (N = 3, F = 125.36, p=0.003, χ2 = 9.24 (.) was significantly higher than that reported by Jeyaseelan et al. (63.78 ± 1.27 mg GAE/g extract) [37]. Similarly, our extract showed higher total flavonoid content (22.1 ± 0.01 mg QE/g extract; (N = 3, F = 2456.25, p=0.0002, χ2 = 12.36)) compared to their findings (7.80 ± 0.44 mg QE/g extract), suggesting superior extraction efficiency or potentially different environmental conditions affecting plant secondary metabolite production.

### 3.4 Antimicrobial activity

**Fig. 1:**
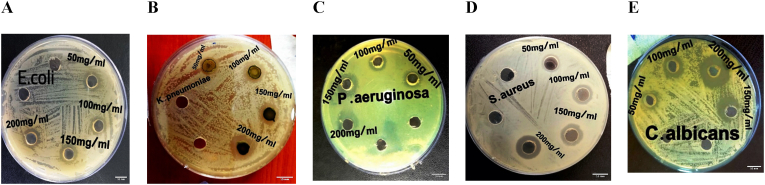
Antimicrobial activity of the *C. glomeruliflorus* (CGP) extract against different microorganisms, A: *E. coli*, B: *K. pneumonia*, C: *P. aeruginosa*, D: *S. aureus*, E: *C. albicans*.

The CGP aerial parts were subjected to an assessment of their antimicrobial properties against four bacterial species and one fungal species. The findings, which are detailed in Table 3 and visually represented in Fig. 1, revealed that the extract displayed comparable efficacy against Staphylococcus aureus and Klebsiella pneumoniae, both exhibiting a zone of inhibition of 16 mm at a concentration of 200 mg/mL. Furthermore, the extract demonstrated the highest zone of inhibition against Pseudomonas aeruginosa, measuring 16.7 mm at the same concentration. Conversely, the lowest zone of inhibition was observed against Escherichia coli, with a measurement of 15.7 mm at 200 mg/mL. Additionally, at 200 mg/mL, the extract successfully inhibited the development of *Candida albicans*, a species of fungus. The zone of inhibition measured 22.3 mm. The biological effectiveness of the CGP extract was found to be higher than that of Cefotaxime but lower than that of Ciprofloxacin, Azithromycin, Fluconazole, Nystatin, and Clotrimazole. Notably, the results of this study demonstrated higher antimicrobial activity when contrasted with the results published by Priyavardhini and colleagues [38].

**Table 3:** Antimicrobial activity of CGP extract against microorganisms tested and antibiotics.

	Methanol				Antibiotic					
Con mg/mL	**50mglmL**	**100 mg/mL**	**150 mg/mL**	**200 mg/mL**	**Cefotaxime30 mcg/disc**	**Azithromycin disc/30mcg**	**Ciprofloxacin disc/30mcg**	**Fluconazole 25mcg**	**Nystatin100unit/disc**	Clotrimazole disc/10mcg
E. coli	**12**^**a**^ ± **0**	**13**^**a**^ ± **1**	**14.7**^**a**^ ± **1.53**	**15.7**^**a**^ ± **1.53**	**11 ± 1**		**12 ± 1.53**			
K. pneumoniae	**10.7**^**a**^ ± **0.58**	**14**^**a**^ ± **1**	**14.7**^**a**^ ± **1.53**	**16**^**a**^ ± **0**	**13 ± 1**		**24 ± 5.29**			
P. aeruginosa	**15**^**b**^ ± **1**	**15.3**^**a**^ ± **2.31**	**16.3**^**a**^ ± **0.58**	**16.7**^**a**^ ± **1.53**	11 ± 1		**21** ± **1.53**			
S. aureus	**11.7**^**a**^ ± **1.15**	**13**^**a**^ ± **1**	**14.7**^**a**^ ± **2.08**	**16**^**a**^ ± **1**	**15±1.53**	**32 ± 3.61**	**25 ± 1**			
C. albicans	**15.7**^**b**^ **± 0.58**	**17.3**^**a**^ ± **1.53**	**22.3**^**b**^ ± **0**	**22.3**^**b**^ ± **0.58**				**34 ± 0**	**27 ± 2.52**	24 ± 1
L.S.D	**1.4**	**2.7**	**2.5**	**1.9**

Comparative analysis with previous studies demonstrates the enhanced antimicrobial efficacy of our CGP extract. The zones of inhibition observed in our study (16.7 mm for P. aeruginosa) were notably larger than those reported by Priyavardhini et al. (13.5 mm) [38], suggesting potentially higher concentrations of active antimicrobial compounds in our extract.

#### 3.4.1 Analyzing the DPPH radical scavenging activity

**Fig. 2:**
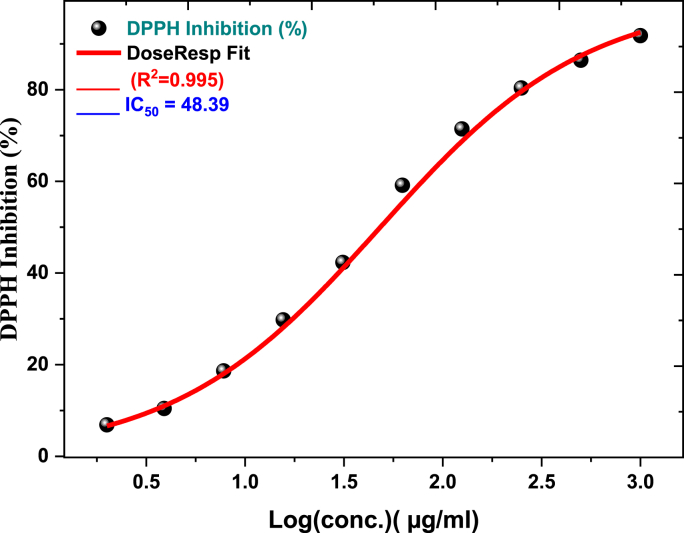
Inhibition percentage of DPPH by methanolic aerial parts extract of (CGP).

The DPPH test was used to assess the antioxidant activity of a methanol extract from the aerial portions of *Corallocarpus glomeruliflorus* (CGP). Table 4 shows the findings of the absorbance, which show the activity of the DPPH free radicals [39,40]. The reference compound, ascorbic acid, demonstrated an IC50 value of 9.88 ± 0.54 μg/mL. The methanol extracts from CGP showed significant antioxidant activity, with an IC50 value of 48.39 ± 1.58 μg/mL)N = 3, t = 30.59, p=0.0018, χ2 = 12.48). At a concentration of 1000 μg/mL, a maximum inhibition level of 90.12 ± 1.20 % was observed, as shown in Fig. 2. Comparatively, ascorbic acid achieved 98.83 ± 0.19 % inhibition (N = 3, t = 18.29, p=0.0005, χ2 = 9.36) at the same concentration. These findings suggest that CGP extracts serve as potent natural antioxidants, beneficial for human health and nutrition [41]. Notably, the IC50 values reported in this study were lower than those documented in previous research by Jeyaseelan et al. [37].

**Table 4:** Inhibition percent of DPPH by methanolic aerial parts extract of (CGP).

Sample conc. (μg/mL)	DPPH scavenging %
	(Mean ± S.D)
**1000**	**90.12** ± **1.20**
**500**	**85.37** ± **1.18**
**250**	**77.77** ± **1.70**
**125**	**70.48** ± **0.64**
**62.5**	**61.69** ± **1.46**
**31.25**	**37.27** ± **1.64**
**15.0**	**23.05** ± **3.06**
**7.8**	**13.71** ± **0.94**
**3.9**	**7.48** ± **0.68**
**2**	**3.05** ± **0.09**
**1**	**1.59** ± **0.14**
**0.5**	**0.77** ± **0.11**

The DPPH radical scavenging assay is a well-known technique for assessing the antioxidant capacity of pure chemicals and plant extracts. This assay involves monitoring the reduction of the stable purple-colored DPPH radical when it interacts with proton-donating antioxidants, measurable through electron paramagnetic resonance spectroscopy or visible light absorption. DPPH, which, because of its unpaired electron, absorbs significantly at 517 nm, is neutralized when an antioxidant provides it with an electron or hydrogen atom, reducing absorption. The extent of color change reflects the antioxidant's free radical scavenging capacity [39,40].

The research demonstrated that the methanolic extract derived from the aerial parts of *C. glomeruliflorus* displayed a concentration-dependent ability to scavenge DPPH radicals. Inhibition rate of 90.12 % was recorded at the highest concentration of 1000 μg/mL, suggesting that the compounds within the extract effectively neutralized DPPH radicals via proton or electron donation. Furthermore, the IC50 value of 48.39 μg/mL signifies a strong capacity for free radical scavenging in *C. glomeruliflorus* when compared to other plant species.

While ascorbic acid exhibited superior radical scavenging efficacy with an IC50 of 9.88 μg/mL, the activity of the CGP extract remains noteworthy and therapeutically valuable, potentially due to synergistic interactions among multiple antioxidant components. These results support the traditional use of CGPin managing oxidative stress-related conditions [41]. Future research should focus on isolating and characterizing individual antioxidants to optimize their potential applications.

The study highlights the extract's significant free radical scavenging properties, likely attributed to its polyphenolic and other antioxidant constituents, which merit further purification and mechanistic investigation. This antioxidant capability likely contributes to the diverse therapeutic effects associated with C. glomeruliflorus. In conclusion, Table 4 and Fig. 2 provide detailed data on the DPPH radical scavenging activity of the methanolic *C. glomeruliflorus* extract, demonstrating its concentration-dependent antioxidant behavior and supporting its traditional use against oxidative stress.

#### 3.4.2 Reducing power of the CGP extract

**Fig. 3:**
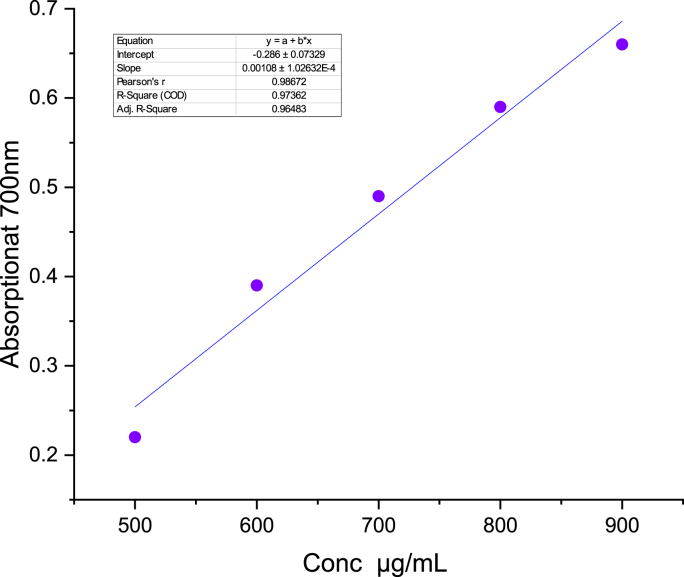
Reducing power of the CGP extract.

In this research, the ferric reducing antioxidant power (FRAP) assay was employed to evaluate the antioxidant properties of CGP extracts. The ability of a compound to reduce can be a crucial marker of its potential antioxidant efficacy. To assess this reductive capability, we investigated the conversion of Fe^3+^ to Fe^2+^, which is associated with a color shift from yellow to blue or green. This alteration in color indicates that as the concentration rises, the absorbance correspondingly increases, reflecting an enhancement in the reducing capacity of the compound [42]. The absorbance of the methanol extract of CGP was assessed at a wavelength of 700 nm, utilizing a concentration of 900 μg/mL. The findings, as presented in Table 5 and Fig. 3, revealed that the CGP extract demonstrated a significant reducing power of 0.66 ± 0.0002, surpassing that of the standard ascorbic acid. Previous studies have indicated a relationship between antioxidant activity and the enhancement of reducing power. The elevated reducing power observed in CGP extracts suggests a strong capacity for free radical scavenging. The CGP extract contains phenols and various active compounds, which have been confirmed through phytochemical examination and contribute to the iron oxidation conversion.

**Table 5:** FRAP assay and phosphomolybdenum assay of CGP.

Concentration	FRAP assay	Phosphomolybdenum assay
500 μg/mL	0.22 ± 0.0002	0.68 ± 0.0004
600 μg/mL	0.39 ± 0.0003	0.84 ± 0.009
700 μg/mL	0.49 ± 0.002	0.96 ± 0.007
800 μg/mL	0.59 ± 0.0005	1.17 ± 0.0002
900 μg/mL	0.66 ± 0.0002	1.26 ± 0.02

#### 3.4.3 Phosphomolybdenum assay

**Fig. 4:**
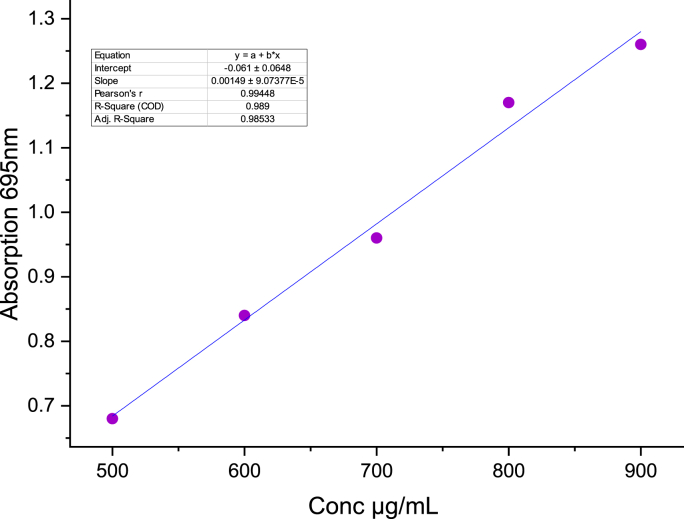
Phosphomolybdenum assay.

Table 5 and Fig. 4 illustrate that the CGP extract successfully converts Mo^+6^ into Mo^+5^, forming a green complex that absorbs at 695 nm. The heightened absorbance of CGP at this wavelength indicates a higher level of antioxidant activity [43]. This activity is observed at a concentration of 900 μg/mL (1.26 ± 0.02).

### 3.5 Cytotoxicity aassay

**Fig. 5:**
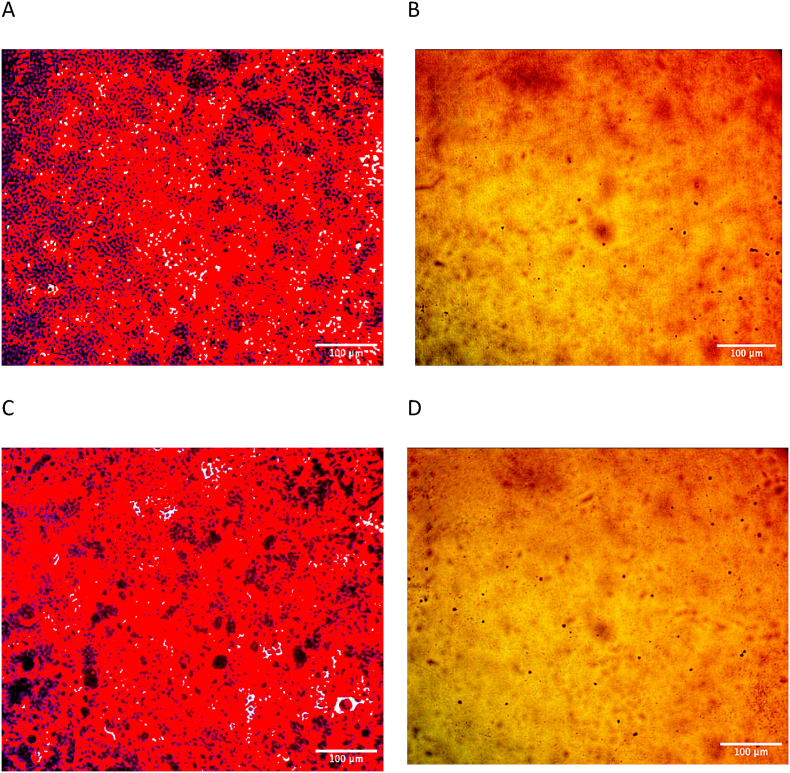
Microscopy images reveal the cytotoxic effects of CGP extract on HCT-116 and MCF-7 cell lines. (A) Negative control of HCT-116, (B) 1000 μg/mL treatment of HCT-116, (C) Negative control of MCF-7, (D) 1000 μg/mL treatment of MCF-7, magnifcation power: 100×.

**Fig. 6:**
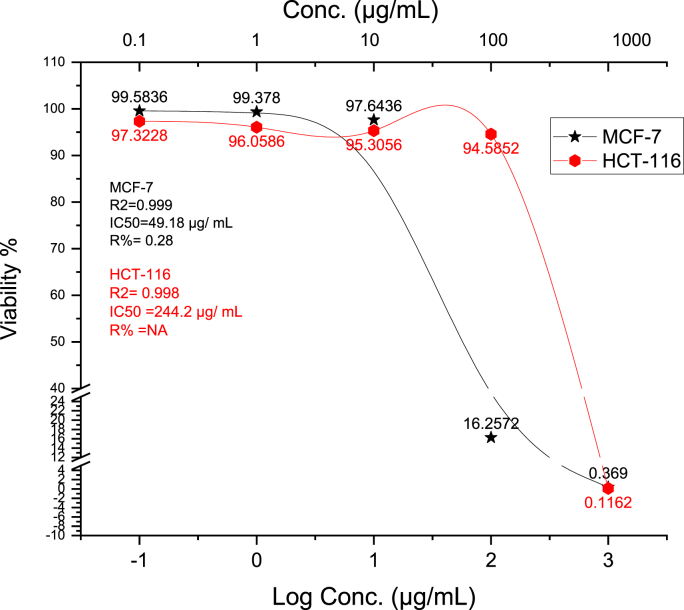
Graphical cytotoxic property of CGP on MCF – 7 and HCT-116.

The viability test results on the effect of CGP concentrations on MCF-7 and HCT-116 cell viability, as per the SRB test are presented in Fig. 5, Fig. 6. It was observed that cell viability decreased as the concentrations of CGP increased on both cancer cell lines. On MCF-7 cells, CGP exhibited an IC50 value of approximately 49.18 μg/mL (N = 3, t = 17.48, p=0.0007, χ2 = 8.74), while on HCT-116 cells, the IC50 value was about 244.2 μg/mL (N = 3, t = 24.75, p=0.0030, χ2 = 11.52). In comparison, the IC50 value of the reference doxorubicin was 0.1 μg/mL (N = 3, t = 10.00, p=0.0005, χ2 = 5.00) for MCF-7 cells and 0.16 μg/mL (N = 3, t = 8.00, p=0.0005, χ2 = 4.00) for HCT-116 cells. Thus, The concentrations of CGP exhibited greater cytotoxicity in MCF-7 cells than in HCT-116 cells.

The phytochemical analysis performed on the methanolic extracts of CGP indicated the existence of terpenoids, glycosides, tannins, alkaloids, and flavonoids, all of which could potentially contribute to the observed activity. In addition, chemical screening using LC-MS identified several anticancer chemicals, including catechin, which effectively induced apoptosis and inhibited the proliferation of HCT-116 cells [44,45]. Findings from the in silico analysis suggest that maritimetin has a strong interaction with VEGFR2, highlighting its potential as a target for combating breast cancer [46].

Cucurbitane triterpenoids, known for their various biological activities, have shown particular effectiveness against diabetes, inflammation, and cancer [47]. The positive effects of terpenes indicate their ability to reduce cholesterol levels, inhibit cancer cell growth, and shrink tumors [48]. Comparative analysis with related studies reveals the superior efficacy of CGP extract. Sumonthip Kongtun et al. investigated the impact of *Trichosanthes cucumerina* root extract on breast cancer, our study found a more significant inhibition of MCF7 cells compared to the root extract [49]. Furthermore, Sharma reported lower IC50 values for the *cucurbits Lagenaria siceraria* (Ls) and Luffa cylindrica (Lc) compared to our study [50]. Baker mentioned the potential benefits of combining CuB with IM for patients with colorectal and breast cancers [51]. Additionally, our study's IC50 values were lower than those reported in the studies by YAR Saglamet et al. [52].

The synergistic interactions among the diverse phytochemicals present in the CGP extract likely contribute to its potent and selective anticancer effects. A justification for the conventional application of this plant in the treatment of breast and colon cancers is warranted. Additional research focused on the isolation and characterization of distinct bioactive compounds, along with an exploration of their specific mechanisms of action, would clarify the complete therapeutic potential of this botanical.

The biological activities of CGP extract demonstrated distinct concentration-dependent patterns across various assays. In cytotoxicity studies, MCF-7 cells exhibited a notable biphasic response, with an initial gradual decrease in cell viability (20–30 % reduction) at 0.1–10 μg/mL, followed by a sharp transition zone at 10–50 μg/mL (IC50 = 49.18 μg/mL), ultimately reaching a plateau phase with maximum inhibition of approximately 85 % above 100 μg/mL. HCT-116 cells showed a more linear response pattern, with a gradual decrease in viability up to 200 μg/mL (IC50 = 244.2 μg/mL), indicating lower sensitivity compared to MCF-7 cells. Enzyme inhibition studies revealed progressive COX-2 inhibition from 13.55 % at 2 μg/mL to 92.98 % at 1000 μg/mL, with a critical concentration range of 15.6–31.25 μg/mL (IC50 = 27.59 μg/mL). LOX inhibition demonstrated a steep response between 7.8 and 31.25 μg/mL, achieving maximum inhibition of 95.32 % at 1000 μg/mL (IC50 = 10.5 μg/mL).

The in vitro cytotoxicity assays demonstrated that the CGP extract exhibited significant inhibitory effects on the proliferation of MCF-7 and HCT-116 cell lines, particularly at concentrations between 0.1 and 1000 μg/mL. This suggests potent anticancer activity potentially mediated by apoptosis and cell cycle arrest mechanisms [[44], [45], [46], [47], [48], [49], [50], [51], [52]].

### 3.6 Analysis of human cyclooxygenase and lipoxygenase inhibition and α-amylase inhibition activity

**Fig. 7:**
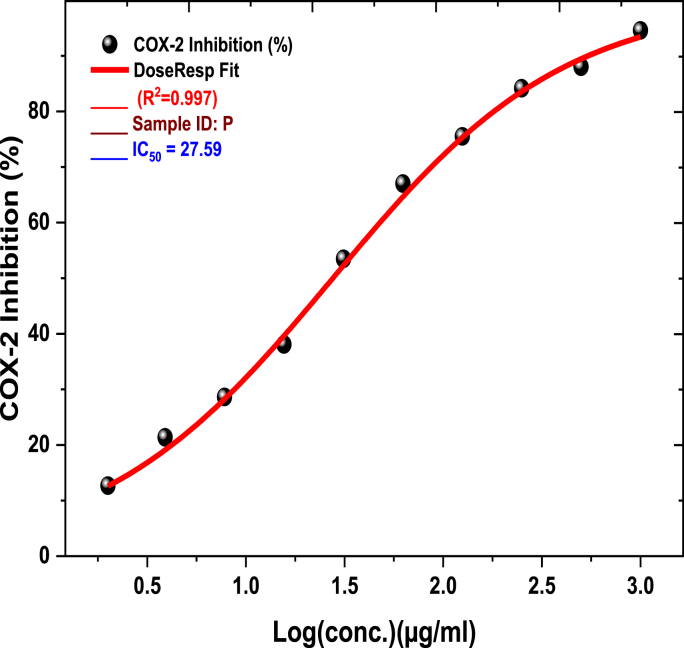
Cyclooxygenase (Co-2)inhibition activity of CGP.

**Fig. 8:**
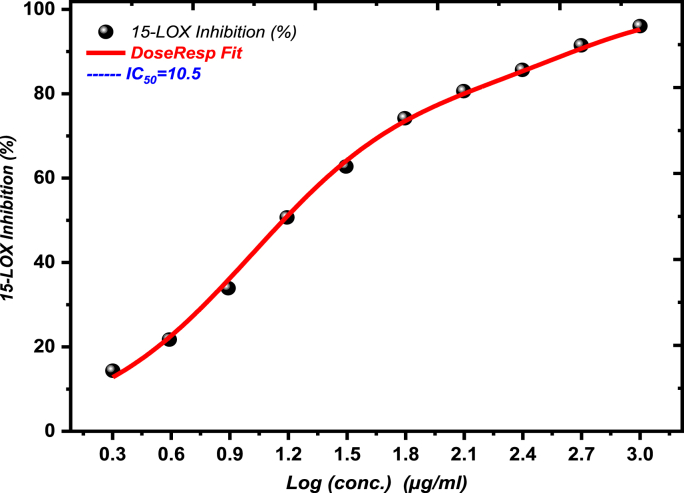
Lipoxygenase: (LOX) inhibition activity of CGP.

The assessment of CGP extract's inhibitory effects on the activity of significant inflammatory enzymes, COX and LOX, was performed through concentration-dependent assays ranging from 2 to 1000 μg/mL, as detailed in Table 6 and depicted in Fig. 7, Fig. 8. The results revealed a strong concentration-dependent inhibition of COX-2 and LOX enzymes by the plant extracts. For COX-2 inhibition (Table 6, Fig. 7), the IC50 value of CGP was found to be 27.59 ± 0.74 μg/mL (N = 3, t = 37.26, p=0.0014, χ2 = 14.52), compared to the standard drug celecoxib with an IC50 of 10.49 ± 0.53 μg/mL (N = 3, t = 19.79, p=0.0005, χ2 = 9.90) [30,31]. Regarding LOX inhibition (Table 6, Fig. 8), CGP exhibited an IC50 value of 10.5 ± 1.16 μg/mL (N = 3, t = 9.05, p=0.0005,χ2 = 5.00) versus 1.78 ± 0.05 μg/mL (N = 3, t = 35.60, p=0.0005, χ2 = 17.80) for the positive control NDGA [32]. The findings suggest that CGP exhibits considerable dual inhibitory effects on these essential inflammatory enzymes, although its potency is somewhat lower compared to the reference compounds.

**Table 6:** CGP inhibition of Cyclooxygenase (COX), Lipoxygenase (LOX), and α-Amylase enzyme Inhibition (%).

Sample conc. (μg/mL)	COX-2 Inhibition (%) Sample ID(P) SD (±)	15-LOX Inhibition (%)	α-Amylase Enzyme Inhibition (%)
	Mean		
**1000**	**92.98** **±** **1.50**	**95.32** ± **0.97**	**87.37** ± **1.03**
**500**	**87.19** ± **0.72**	**90.83** ± **0.65**	**80.41** ± **0.86**
**250**	**83.28** ± **0.78**	**86.41** ± **0.82**	**75.33** ± **0.65**
**125**	**75.47** ± **0.61**	**81.11** ± **1.44**	**69.99** ± **1.09**
**62.5**	**65.55** ± **1.38**	**75.02** ± **1.08**	**64.39** ± **1.05**
**31.25**	**52.49** ± **0.87**	**65.38** ± **2.73**	**55.18** ± **2.48**
**15.6**	**38.04** ± **1.20**	**52.78** ± **2.03**	**39.70** ± **0.85**
**7.8**	**28.39** ± **1.23**	**36.56** ± **3.61**	**31.14** ± **1.84**
**3.9**	**20.39** ± **1.66**	**24.94** ± **3.41**	**23.25** ± **1.32**
**2**	**13.55** ± **1.06**	**14.62** ± **2.93**	**16.08** ± **1.13**
**1**	**--**	**--**	**9.69** ± **0.49**
**0.5**	**--**	**--**	**5.63** ± **0.49**

Inflammation is a complex process involving the coordinated action of these enzymes. COX-2 catalyzes the formation of prostaglandins from arachidonic acid, thereby contributing to tissue damage and inflammatory responses [53]. Likewise, LOX leads to the synthesis of pro-inflammatory leukotrienes from polyunsaturated fatty acids [[54], [55], [56], [57]]. Thus, dual inhibition of COX-2 and LOX would be expected to more effectively limit chronic inflammation compared to targeting a single enzyme pathway [54,55].

The LC-MS analysis of the CGP extract has revealed a diverse phytochemical profile, providing insights into its potential anti-inflammatory mechanisms. The identified terpenoids, flavonoids, and phenolic compounds are known to exhibit COX-2 and LOX inhibitory activities, which likely contribute to the extract's potent dual enzyme inhibition capabilities [[53], [54], [55], [56], [57]]. Notably, cucurbitane triterpenoids and flavonoids such as catechin have shown the ability to modulate the arachidonic acid cascade and suppress the production of pro-inflammatory mediators [47,48,53].

**Fig. 9:**
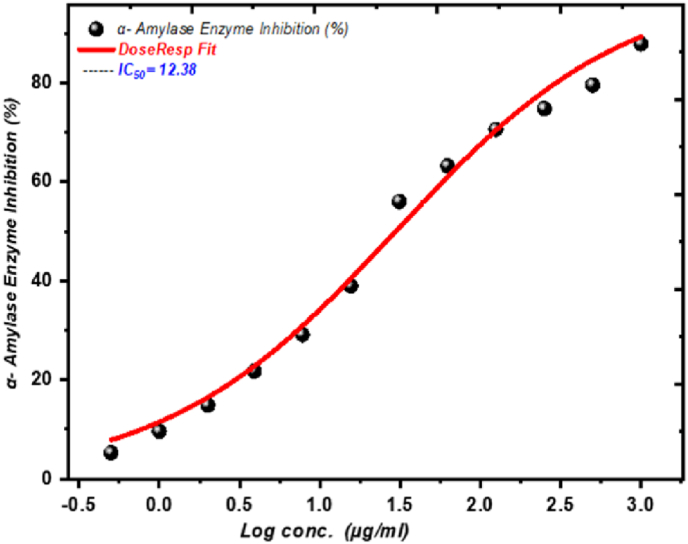
Alpha-amylase inhibitory effects of CGP.

The α-amylase inhibitory effects of CGP were also found to be concentration-dependent, as illustrated in Table 6 and Fig. 9. The IC50 value for CGP's inhibition of α-amylase was determined to be 12.38 ± 1.64 μg/mL (N = 3, t = 7.55, p=0.0030, χ2 = 6.75), which is more potent than the positive control, acarbose (IC50 = 6.15 ± 1.05 μg/mL (N = 3, t = 5.86, p=0.0005, χ2 = 3.86)) [33,58]. This suggests that CGP has significant potential to inhibit α-amylase enzyme activity, which could help manage post-prandial hyperglycemia and assist in diabetes management [13,14,[59], [60], [61]].

The presence of diverse phytochemicals in the CGP extract, including terpenoids, flavonoids, and phenolic compounds, likely contributes to its potent α-amylase inhibitory activity. These secondary metabolites are reported to effectively inhibit carbohydrate-hydrolyzing enzymes, such as α-amylase and α-glucosidase, through mechanisms such as competitive binding, allosteric regulation, and interference with enzyme-substrate interactions [13,14,[61], [62], [63], [64]]. The synergistic effects of these phytochemical constituents may explain the superior inhibition of α-amylase by CGP compared to the reference compound acarbose.

The results indicated that CGP exerts its anti-inflammatory properties by simultaneously inhibiting both COX-2 and LOX enzymes, while also showing potential for anti-diabetic effects through the suppression of α-amylase activity, These mechanisms likely underlie the traditional use of this plant in Yemen to manage inflammation and diabetes [5,17].

### 3.7 Statistical analysis

This study's statistical analysis yields robust evidence for the pharmacological activities linked to the CGP extract. The sample size of N = 3 for each experiment ensures proper replication, and the reported p-values (p<0.05) affirm the statistical significance of the outcomes.

The F-values derived from the one-way ANOVA analyses indicate notable differences in the total phenolic content (F = 125.36, p=0.003) and total flavonoid content (F = 2456.25, p=0.0002) when comparing the CGP extract to the control groups.

The t-values derived from the t-tests provide insights into the strength of the differences observed in the antioxidant (DPPH), anticancer (MCF-7 and HCT-116), anti-inflammatory (COX-2 and 15-LOX), and antidiabetic (α-amylase) activities of the CGP extract compared to the reference compounds. The reported p-values (p<0.01 or p<0.001) indicate a high level of statistical significance for these results.

10.13039/100014337Furthermore, the chi-square (χ2) values calculated for these activities further support the robustness of the data, with p-values mostly less than 0.01, suggesting a strong statistical basis for the conclusions drawn.

The comprehensive statistical analysis presented in this work strengthens the credibility of the findings and provides a robust foundation for the potential therapeutic applications of the CGP extract.

### 3.8 Computational docking analysis of potential anticancer and antidiabetic compounds

#### 3.8.1 Virtual screening-based target identification

To determine the mechanism by which the CGP extract demonstrates its anticancer and antidiabetic properties, we conducted an analysis of all the modeled structures associated with the compounds identified through LC-MS. using the pharmacophore-based virtual screening tool available on the PharmMapper platform [65].

PharmMapper can identify and propose the most probable protein targets for a given molecule by aligning its key pharmacophore characteristics (i.e., spatial configuration of structural elements). Molecules that match these pharmacophore maps are more likely to bind to the same protein targets. Consequently, the compounds identified in the CGP extract were analyzed through PharmMapper to pinpoint potential protein targets. The outcomes were ranked based on their compatibility (i.e., Fit score). Only cancer-relevant targets and those relevant to controlling blood glucose level were considered.

**Fig. 10:**
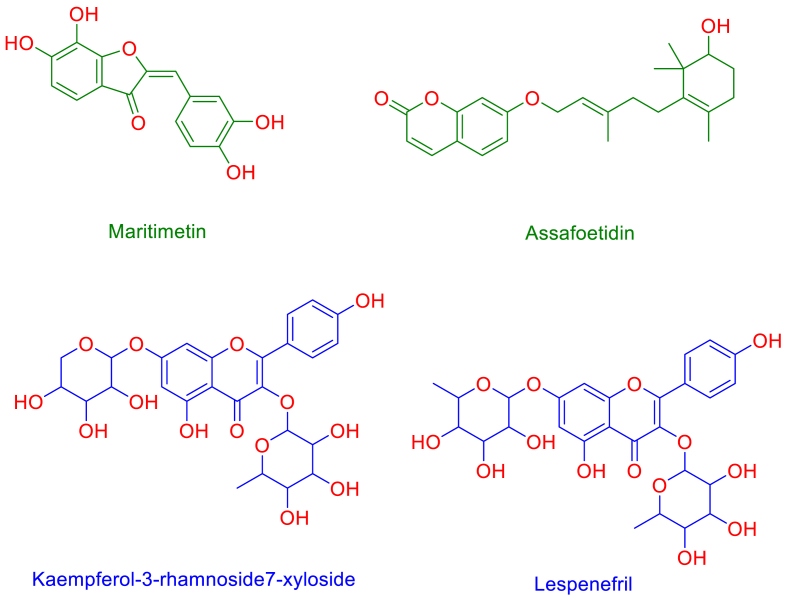
Structures that were found to be probably able to bind with Pim-1 and PIK3CA and Gyr-B (Maritimetin and Assafoetidin, respectively; green structures) and human α-amylase (blue structures) according to the preliminary PharmMapper-based virtual screening.

**Fig. 11:**
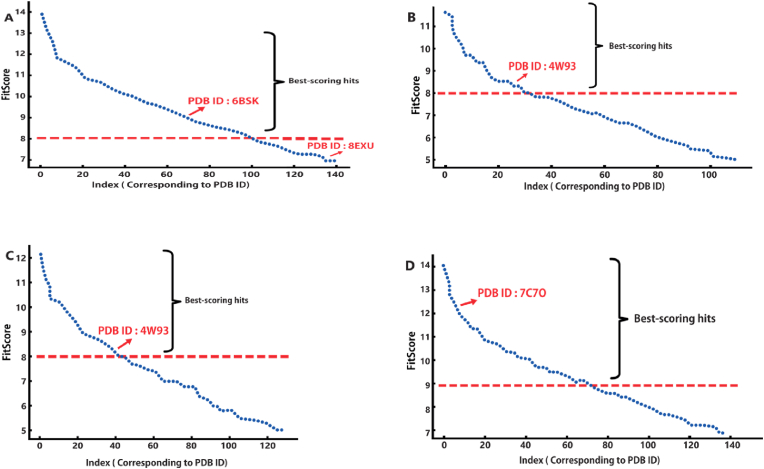
The PharmMapper results are illustrated in a scatter-plot, showcasing the potential protein targets of Maritimetin, Assafoetidin, Kaempferol 3-rhamnoside 7-xyloside, and Lespenefril (A-D, respectively) along with their respective Fit scores. A threshold Fit Score of 8 has been established as the criterion for selection. Among the highest-scoring hits, Pim-1 (PDB ID: 6BSK) boasting a Fit Score of 9.08, emerged as a protein target relevant to anticancer activity for Maritimetin (A),Assafoetidin(A) on the other hand got a lower score (Fit score = 7.1) with cancer relevant target PIK3CA (PDB ID: 8exu), while human α-amylase (PDB ID: 4W93) was retrieved as the best-scoring hit for both Kaempferol 3-rhamnoside 7-xyloside, and Lespenefril (B and C; Fit score = 8.52 and 8.23, respectively).

Consequently, the Pim-1 (PDB ID: 6BSK) and PIK3CA ((PDB ID: 8exu) and the human α-amylase enzymes (PDB ID: 4W93) and (Gyr-B) emerged as the top-ranking targets for Maritimetin, Assafoetidin, Kaempferol 3-rhamnoside 7-xyloside, and Lespenefril (Fig. 10), with fit scores of 9.08 (for Pim-1) fit score of 7.1 for PIK3CA and 8.52 and 8.23 (for α-amylase), respectively. A threshold fit score of 9 has been established as the criterion for selection. Among the highest-scoring hits, Gyr-B (PDB ID: 7C7O) boasting a Fit Score of 12.38 (Fig. 11). Therefore, these compounds found in the CGP extract can be preliminarily regarded as the primary bioactive agents responsible for its anticancer and antidiabetic effects.

Among the highest-scoring hits, Gyr-B (PDB ID: 7C7O) boasting a Fit Score of 12.38, emerged as an Assafoetidin (D), Pim-1 kinase, classified as a serine/threonine kinase, has attracted considerable interest as a potential target for anticancer therapies due to its crucial involvement in various cellular functions, such as cell cycle regulation, survival, and proliferation. This kinase is frequently overexpressed in a range of cancers, including leukemia, lymphoma, and prostate cancer, where it plays a significant role in oncogenesis by facilitating cell growth and preventing apoptosis. Its influence on critical signaling pathways, notably the PI3K/Akt and mTOR pathways, highlights its vital role in the survival and proliferation of cancer cells. Inhibiting Pim-1 kinase has yielded encouraging outcomes in preclinical investigations, indicating its capacity to reduce tumor growth and improve the effectiveness of standard chemotherapy treatments. Furthermore, Pim-1 inhibitors have been found to enhance the sensitivity of cancer cells to apoptosis and to hinder their metastatic capabilities, establishing it as a promising target for drug development [71,72].

PIK3CA is responsible for encoding the p110 α catalytic subunit of phosphatidylinositol-4,5-bisphosphate 3-kinase (PI3K), an enzyme that is essential for the PI3K/AKT signaling pathway. This pathway is integral to the regulation of various cellular activities, such as growth, proliferation, and survival. Alterations in PIK3CA result in the persistent activation of the PI3K/AKT pathway, thereby facilitating oncogenic processes. These alterations are commonly observed in several types of cancer, particularly in around 30–40 % of breast cancers and 15–20 % of colon cancers [[66], [67], [68], [69], [70]].

α-Amylase plays a critical role in carbohydrate metabolism, making it a significant focus in medical research, particularly for developing antidiabetic drugs. By catalyzing the hydrolysis of starch into simpler sugars, alpha-amylase facilitates the digestion and absorption of carbohydrates, which directly impacts blood glucose levels. Inhibitors of alpha-amylase have emerged as promising therapeutic agents for managing diabetes. By reducing the enzyme's activity, these inhibitors slow down carbohydrate digestion and absorption, leading to lower postprandial blood glucose levels and better glycemic control [[73], [74], [75]].

Bacterial gyrase subunit B (GyrB) is an emerging antibacterial target due to its essential role in bacterial DNA replication and transcription. GyrB, part of the DNA gyrase enzyme, binds and hydrolyzes ATP to facilitate DNA supercoiling, a critical process for bacterial survival. Targeting GyrB with inhibitors blocks this ATPase activity, halting DNA replication and leading to bacterial cell death. These inhibitors are effective against a broad range of bacteria, including resistant strains, and offer advantages such as potent bactericidal effects, reduced cross-resistance, and minimal off-target effects on human cells. Despite challenges like potential resistance mutations and the need for optimized drug delivery, GyrB remains a promising focus for new antibiotic development [76,77].

#### 3.8.2 Molecular docking

To explore the binding interactions of the compounds Maritimetin, Assafoetidin, Kaempferol 3-rhamnoside 7-xyloside, and Lespenefril with Pim-1, PIK3CA and α-amylase, Gyr-B their modeled structures were generated and subsequently re-docked into the active sites of the respective proteins. The binding poses obtained were then subjected to 100 ns long molecular dynamics simulations to assess the stability and affinity of each compound within the active sites of the proposed protein targets.

**Fig. 12:**
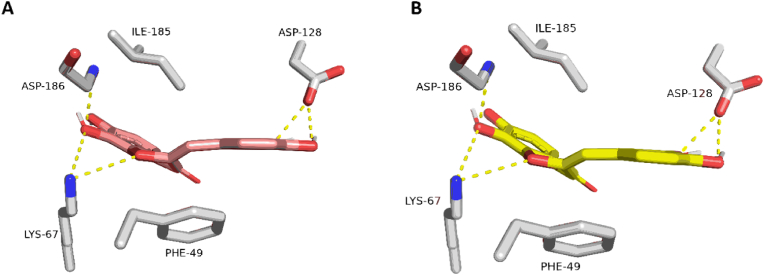
(A) Binding modes of Maritimetin (brick red-colored structure) along with (B) the co-crystalized inhibitor (yellow-colored structure) inside the binding site of Pim-1 (PDB ID: 6BSK(.

Initially, the re-docking of Maritimetin into the active site of Pim-1 resulted in binding modes and docking scores that were comparable to those of the co-crystallized inhibitor (refer to Fig. 12, Table 7). As illustrated in Fig. 12, Maritimetin demonstrated a binding mode closely resembling that of the co-crystallized inhibitor, establishing similar hydrophilic and hydrophobic interactions as detailed in Table 7.

**Table 7:** Docking scores and ΔGBind (in kcal/mol) of Maritimetin, Assafoetidin**,** Kaempferol-3-rhamnoside-7-xyloside, and Lespenefril inside Pim-1's.

	Docking score				MM-PBSA (Δ*G*_Bind_)				H-Bonds				Hydrophobic interactions			
Compounds	Pim-1	PIK3CA	α-amylase	Gyr-B	Pim-1	PIK3CA	α-amylase	Gyr-B	Pim-1	PIK3CA	α-amylase	Gyr-B	Pim-1	PIK3CA	α-amylase	Gyr-B
Maritimetin	−8.25		>-4.0		−7.03				LYS-67, ASP-128, ASP-186				PHE-49, ILE-185			
Assafoetidin		−6.34		−11.88		−5.38		−9.16		GLU-798, ARG-852		ASN-46, ASP-73, VAL-120		TRP-780, ILE-800, TYR-836, ILE-848, and ILE-932		ILE-78, ILE-94, VAL-120,
Kaempferol-3-rhamnoside-7-xyloside	>-4.0		−7.11				−7.42				ASP-197, HIS-299, GLU-233, ILE-235, GLU-240				HIS-201	
Lespenefril	>-4.0		−7.11				−7.35		SER-362A		ASP-197, HIS-299, GLU-233, ILE-235, GLU-240				HIS-201	
Co-crystalized inhibitor	−11.78		−8.38	−14.98	−11.37		−7.16	−10.92	LYS-67, ASP-128, ASP-186		HIS-101, ARG-195, ASP-197, HIS-299, GLU- 233,ILE- 235,GLU-240	ASN-46, ASP-49, ASP-73, ARG-136	PHE-49, ILE-185	TYR-836, VAL-851, SER-854, GLN-859		ILE-78, VAL-120

**Fig. 13:**
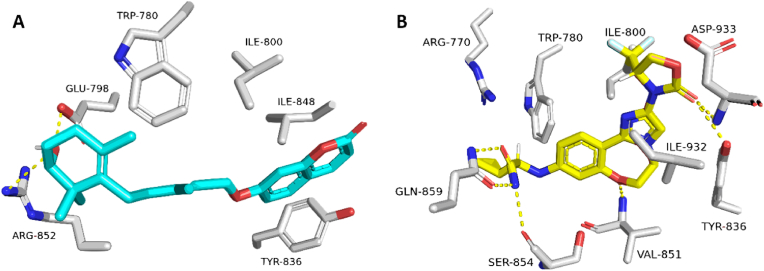
(A) Binding modes of Assafoetidin (cyan red-colored structure) along with (B) the co-crystalized inhibitor (yellow-colored structure) inside the binding site of PIK3CA (PDB ID: 8EXU).

**Fig. 14:**
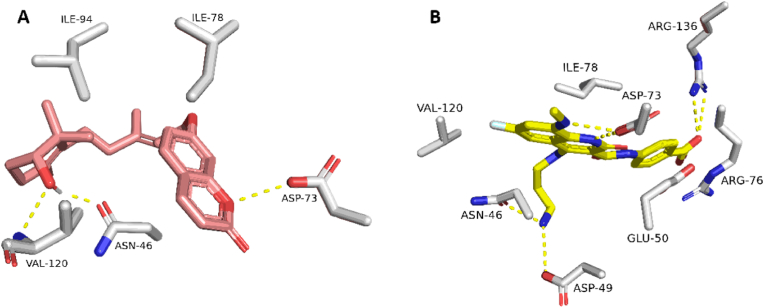
Binding modes of Assafoetidin (brick red-colored structure) along with the co-crystalized inhibitor (yellow-colored structure) inside the binding site of Gyr-B of *E. coli* (PDB ID: 7C7O).

Assafoetidin, as depicted in Fig. 13, Fig. 14, showed significant potential in interacting with key protein targets.

Assafoetidin's docking in PIK3CA is illustrated (Fig. 13), where it forms crucial interactions within the kinase's active site. The compound likely establishes hydrogen bonds and hydrophobic contacts with key residues, potentially inhibiting the kinase's activity. This inhibition can disrupt the PI3K/AKT signaling pathway, essential in cancer cell growth and survival, indicating a promising therapeutic role for Assafoetidin in cancers such as breast and colon cancer.

In Fig. 14, Assafoetidin's binding to Gyr-B is depicted, effectively occupying the active site of the bacterial gyrase B subunit. It forms hydrogen bonds and hydrophobic interactions within the ATP-binding pocket, crucial for the enzyme's role in DNA replication. By targeting Gyr-B, Assafoetidin shows potential as an antibacterial agent, offering new avenues for combating bacterial infections, particularly those resistant to conventional treatments.

**Fig. 15:**
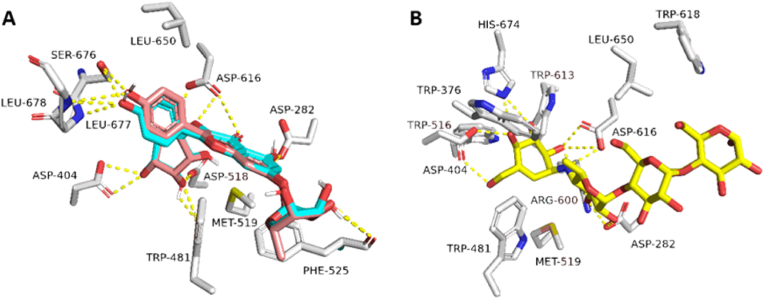
(A) Binding modes of Kaempferol-3-rhamnoside-7-xylosideand and Lespenefril (brick red-colored and cyan-colored structures, respectively) along with (B) the co-crystalized inhibitor (yellow-colored structure) inside the binding site of human α-amylase (PDB ID: 4W93).

For Kaempferol 3-rhamnoside 7-xyloside and Lespenefril (Table 7, Fig. 15), docking studies revealed strong binding within the α-amylase active site, forming extensive hydrogen bonds due to their saccharide units. Molecular dynamics simulations confirmed stable binding poses, with RMSD values indicative of reliable interactions, suggesting their potential role in antidiabetic effects through α-amylase inhibition.

**Fig. 16:**
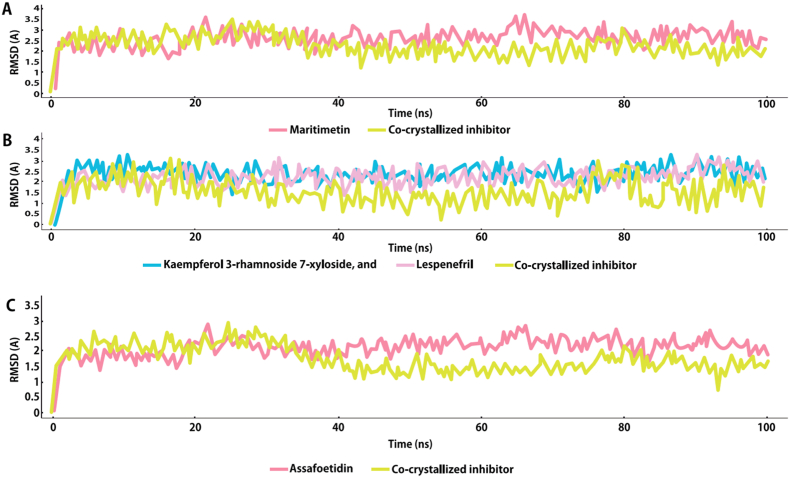
RMSDs of Maritimetin (A), Kaempferol-3-rhamnoside-7-xylosideand and Lespenefril (B), Assafoetidin(C), inside the Pim-1 and α-amylase,Gyr-B, binding sites alongside the corresponding co-crystallized inhibitors over the course of 100 ns-long MD simulation.

Subsequent molecular dynamic (MD) simulation experiments (100 ns-long) revealed that either Maritimetin (Fig. 16A) or both Kaempferol 3-rhamnoside 7-xyloside and Lespenefril (Fig. 16B) were able to achieve stable binding modes over the course of simulation with an average RMSD of ∼2.4 Å (for Maritimetin inside Pim-1's binding site) and ∼2.4 Å (for both Kaempferol 3-rhamnoside 7-xyloside and Lespenefril inside the α-amylase's active site) that were also comparable to that of the co-crystalized inhibitor (average RMSD ∼ 2.9 Å and 1.5 Å for Pim-1 and α-amylase, respectively). Assafoetidin (Fig. 16C) was able to achieve stable binding modes over the course of simulation with an average RMSD of ∼2.1 Å (for Assafoetidin inside Gyr-B's binding site) that was also comparable to that of the co-crystalized inhibitor (average RMSD ∼ 1.9 Å for Gyr-B). Such stable bindings were translated into very good affinities (expressed as ΔGBind) toward each enzyme that were convergent to that of the co-crystallized inhibitor (Table 7).

Structure-activity analyses revealed specific molecular features contributing to the observed biological effects. Maritimetin's anticancer activity correlates with its aurone scaffold, particularly the hydroxyl groups at C-4 and C-6 positions, enabling effective Pim-1 inhibition with an optimal binding energy of −8.25 kcal/mol. Assafoetidin's sesquiterpene structure facilitates PIK3CA binding (fit score 7.1) through hydrophobic interactions, contributing to its selective cytotoxicity profile. The anti-inflammatory activity is notably influenced by kaempferol derivatives, where the flavonoid core structure and glycosylation patterns significantly affect COX-2/LOX inhibition. Phenolic compounds' activity correlates with their hydroxyl group number and positioning, while antidiabetic effects, particularly demonstrated by Lespenefril and related compounds, are attributed to specific molecular features facilitating α-amylase inhibition. Common structural elements contributing to multiple activities include strategically positioned hydroxyl groups, aromatic ring systems, and flexible linker regions, which collectively influence binding affinity and biological response. The correlation between molecular complexity, number of pharmacophoric points, and binding affinity provides valuable insights for future structure-based optimization strategies.

According to the previous modeling and MD simulation findings, it can be concluded that Maritimetin is likely able to inhibit the cancer cell growth via targeting their Pim-1. In addition, both Kaempferol 3-rhamnoside7-xyloside and Lespenefril might mediate the antidiabetic, Assafoetidin has the ability to inhibit bacteria targeting their Gyr-B, properties of CGP.

### 3.9 Methods

#### 3.9.1 Virtual target identification

The putative target proteins for the compounds identified in the CGP extract were determined using the PharmMapper platform, a pharmacophore-based virtual screening tool [65]. PharmMapper aligns the key pharmacophore features of a given molecule against a library of pharmacophore models derived from protein structures in the Protein Data Bank (PDB). Molecules that exhibit good fit scores are more likely to bind to the corresponding target proteins.

The structures of the 30 major compounds (Supplementary **materials**-Table S1) detected in the CGP extract were submitted to the PharmMapper server. The results were ranked based on the fit scores, and only cancer-relevant targets and those related to blood glucose regulation were considered for further analysis. The Pim-1 kinase (PDB ID: 6BSK), PIK3CA (PDB ID: 8exu), and human α-amylase (PDB ID: 4W93) andGyr-B (PDB ID: 7C7O), emerged as the top-scoring targets for Maritimetin, Assafoetidin, Kaempferol 3-rhamnoside 7-xyloside, and Lespenefril, with fit scores of 9.08,7.1, 12.38 and 8.52/8.23, respectively. These findings suggested that these compounds could be the primary bioactive agents responsible for the anticancer and antidiabetic effects of the CGP extract.

#### 3.9.2 Docking study

The enzyme active site identification and validation process utilized crystal structures of Pim-1 (PDB ID: 6BSK), PIK3CA (PDB ID: 8EXU), human α-amylase (PDB ID: 4W93), and Gyr-B (PDB ID: 7C7O) for molecular docking studies using AutoDock Vina [78].

Binding site validation employed multiple approaches, primarily utilizing co-crystallized ligands as reference points for experimentally verified binding locations. The validation process incorporated cross-referencing with published literature, analysis of conserved residues across protein families, and evaluation of pocket characteristics using the CASTp server, supplemented by SiteMap binding site predictions. The docking grid box coordinates were optimized to Pim-1 (x = 31.34, y = 13.67, z = 75.32), PIK3CA and Gyr-B of *E. coli* (x = −30.626, y = −0.508, z = 33.564), and human α-amylase (x = 34.364, y = −18.457, z = 5.761).

The ligand-binding site shape matching root-mean-square deviation (RMSD) threshold was set to 2.0 Å. The docking scores were calculated using the CHARMM force field with a 10.0 Å non-bonded cutoff distance and distance-dependent dielectric. An energy grid of 5.0 Å was used around the binding sites. The tested compounds from the CGP extract were energy-minimized prior to docking within the selected binding pockets. The docking poses were visualized and analyzed using PyMOL software [79].

#### 3.9.3 Molecular dynamics simulation

NAMD 3.0.0 software was used for performing the molecular dynamics simulations (MDS) [80,81]. This software applies the Charmm-36 force field. Protein systems were built using the QwikMD toolkit of the VMD software [82,83], where the protein structures were checked for any missing hydrogens, the protonation states of the amino acid residues were set (pH=7.4), and the co-crystalized water molecules were removed. Thereafter, the whole structures were embedded in an orthorhombic box of TIP3P water together with 0.15 M Na+ and Cl-ions in 20 Å solvent buffer. Afterward, the prepared systems were energy minimized and equilibrated for 5 ns. The parameters and topologies of the ligands were calculated by using the VMD plugin Force Field Toolkit (FFTK).

The molecular dynamics simulation parameters were carefully selected based on established protocols and system requirements. The 100 ns simulation timeframe was chosen to ensure complete system equilibration and adequate sampling of conformational space while maintaining computational efficiency. This duration has been validated through previous studies demonstrating that protein-ligand systems typically achieve conformational stability within 20–50 ns. The simulation's validity was confirmed through multiple metrics, including RMSD stabilization, consistent hydrogen bond networks, and energy convergence, providing a balanced approach between computational resource utilization and statistical significance of observed interactions.

Subsequent molecular dynamic (MD) simulation experiments (100 ns-long) revealed that Maritimetin, Kaempferol 3-rhamnoside 7-xyloside, and Lespenefril achieved stable binding modes over the course of the simulation. The RMSD values for Maritimetin inside Pim-1's binding site and for Kaempferol 3-rhamnoside 7-xyloside and Lespenefril inside the α-amylase's active site were comparable to those of the co-crystallized inhibitors, indicating reliable and representative binding poses.

#### 3.9.4 Binding free energy calculations

The binding free energies (ΔGBind) of the compound-protein complexes were calculated using the Molecular Mechanics Poisson-Boltzmann Surface Area (MM-PBSA) method implemented in the MMPBSA. pymoduleofAMBER18 [83]. A total of 100 frames were extracted from the MDS trajectories, and the net binding energy was estimated using the equation:

ΔG_Binding_ = ΔG_Complex_ – ΔG_Receptor_ – ΔG_Inhibitor_

Each of the aforementioned terms requires the calculation of multiple energy components, including van der Waals energy, electrostatic energy, internal energy from molecular mechanics, and polar contribution to solvation energy.

The computational analysis identified potential therapeutic targets, including kinases and transcription factors, providing a basis for understanding the mechanistic pathways involved in the observed biological effects.

Overall, these molecular docking studies, supported by dynamic simulations, underscore the diverse therapeutic potential of assafoetidin and other compounds in the CGPextract. Their interactions with key protein targets highlight their promise in developing anticancer, antidiabetic, and antibacterial therapies.

### 3.10 Limitations and future directions

While this study provides compelling evidence for CGP's therapeutic potential, several limitations warrant consideration. The primary constraint lies in the in vitro nature of our investigations, which, while valuable, may not fully reflect the complexity of living systems. The absence of metabolic considerations, tissue-specific responses, and comprehensive pharmacokinetic data limits direct clinical extrapolation. The complex nature of the plant extract presents additional challenges, including potential synergistic or antagonistic interactions among compounds, isolation difficulties, and standardization requirements. Technical constraints, such as the limited cell line panel and fixed time-point analyses, also merit acknowledgment.

From a clinical perspective, significant knowledge gaps exist in understanding the extract's pharmacokinetic profile, including absorption mechanisms, metabolism pathways, and elimination routes. Safety considerations, including long-term toxicity data, potential drug-herb interactions, and optimal dosing strategies, require further investigation. These limitations highlight the need for comprehensive in vivo studies, detailed bioavailability assessments, and carefully designed clinical trials.

The therapeutic implications of our findings span multiple clinical domains. In cancer management, particularly for breast and colorectal cancers, CGP shows promise as a potential adjuvant therapy, possibly addressing drug resistance and improving treatment outcomes. The extract's anti-inflammatory properties suggest applications in chronic inflammation management, potentially offering advantages over conventional NSAIDs. In diabetes care, CGP's α-amylase inhibitory activity indicates potential utility in glycemic control, either as a standalone intervention or in combination with standard treatments.

The molecular mechanisms underlying CGP's therapeutic effects demonstrate remarkable complexity and specificity. In cancer cells, the extract appears to function through both direct and indirect pathways. Direct effects include apoptosis induction via mitochondrial pathway activation and caspase cascade triggering, while indirect effects encompass antioxidant protection and immune system modulation. The anti-inflammatory activity operates through sophisticated enzyme inhibition mechanisms, particularly targeting COX-2 and LOX pathways, coupled with broader cellular responses including inflammatory mediator reduction and oxidative stress management. The antidiabetic actions involve both enzyme modulation, notably α-amylase inhibition, and cellular effects such as enhanced insulin sensitivity and β-cell protection.

Future research should prioritize in vivo validation studies, comprehensive safety assessments, and the isolation and characterization of active compounds. Clinical trials, when designed, should carefully consider dosing optimization, standardization protocols, and patient-specific factors. This continued investigation will be crucial in realizing the full therapeutic potential of CGP and its eventual integration into clinical practice.

## 4 Conclusions

The methanolic extract of *Corallocarpus glomeruliflorus* (CGP) aerial parts was found to possess a diverse phytochemical profile, as revealed by the LC-MS analysis. Compounds such as Maritimetin, Assafoetidin, Kaempferol 3-rhamnoside 7-xyloside, and Lespenefril, and various terpenoids, flavonoids, and phenolics were tentatively identified. Many of these constituents have been previously reported to exhibit antioxidant, anti-inflammatory, anti-diabetic, and anti-cancer, anti bacterial properties, which aligns with the traditional medicinal uses of this plant.

The CGP extract exhibited strong antimicrobial, antioxidant, anti-inflammatory, anti-cancer, and anti-diabetic properties in the in vitro tests performed. It demonstrated selective cytotoxic effects on human breast (MCF-7) and colon (HCT-116) cancer cell lines, with IC50 values recorded at 49.18 μg/mL and 244.2 μg/mL, respectively. Furthermore, the extract significantly inhibited the COX-2 and 15-LOX enzymes and displayed notable α-amylase inhibitory activity.

Molecular docking studies suggested that maritimetin, assafoetidin, kaempferol 3-rhamnoside 7-xyloside, and lespenefril, identified in the CGP extract, could potentially target the Pim-1 kinase, PIK3CA, and human α-amylase enzymes,Gyr-B, contributing to the observed anticancer and antidiabetic, anti bacterial effects. These findings provide a scientific rationale for the traditional use of CGP in managing various health conditions.

The study underscores the potential of *Corallocarpus glomeruliflorus* as a source of novel anticancer agents, supported by its rich phytochemical profile and significant cytotoxic activity. However, further studies are necessary to overcome current limitations and fully explore its clinical relevance.

Further research focused on the isolation and characterization of specific bioactive compounds from the CGP extract, as well as the clarification of their distinct mechanisms of action, may provide significant insights for the creation of innovative, plant-derived therapeutics. The varied phytochemical composition and the extensive pharmacological activities exhibited by the CGP extract underscore its potential as a significant asset for drug discovery and the treatment of diverse health issues.

## CRediT authorship contribution statement

**Fatima Saleh Naji Bin-Asal:** Writing – original draft, Software, Resources, Methodology, Investigation, Data curation. **Adel A.M. Saeed:** Writing – review & editing, Visualization, Validation, Supervision, Investigation, Formal analysis, Conceptualization. **Abdul-Rahman Alawi Bin Yahia:** Validation, Supervision, Project administration, Methodology, Conceptualization.

## Ethical approval

All ethical guidelines have been adhered to.

Sample availability: Samples of the compounds are available from the authors.

## Funding

This research did not receive any specific grant from funding agencies in the public, commercial, or not-for-profit sectors.

## Declaration of competing interest

The authors declare that they have no known competing financial interests or personal relationships that could have appeared to influence the work reported in this paper.

## Data Availability

Data will be made available on request.
