# Supplementary material for: Corallocarpus glomeruliflorus: Pharmacological potential revealed by phytochemical and in silico investigations
Source: Biochem Biophys Rep. 2025 Feb 7;41:101940. doi: 10.1016/j.bbrep.2025.101940 (PMC11848803; doi:10.1016/j.bbrep.2025.101940)
Supplement: Multimedia component 1 [file mmc1.docx]

**Supplementary Materials**

***Determination of total phenols content (TPC) and total flavonoid content (TFC)***

**Figure S1: A calibration curve of Gallic acid**

**Figure S2 : A calibration curve of Quercetin**

The CGP extract has a total phenolic content (TPC) of 88.12±4.48 mg GAE/g extract (N=3, F=125.36, p=0.003, χ2=9.24(. The methanolic extract of CGP had a higher total flavonoid concentration (22.1±0.01 mg QE/g extract (N=3, F=2456.25, p=0.0002, χ2=12.36().

Table S1: Bioactive compounds identified in methanolic extract of GCP aerial parts by LC-MS (-ve,+ve mode)

| **Biological activity** | **Polarity** | **m/z** | **RT** | **Mass** | **Compound structure** | **Formula** | **Name** | **NO.** |
| --- | --- | --- | --- | --- | --- | --- | --- | --- |
| **It is used to regulate plant growth and reduce bud elongation** | **Positive** | **404.1538** | **8.102** | **381.1649** |  | **C_16_H_23_N_5_O_6_** | **Cis-zeatin-O-glucoside** | **1** |
| **Antioxidant, anti-cancer[1]** | **Positive** | **169.0749** | **8.606** | **168.0679** | **** | **C_11_H_8_N_2_** | **Norharman** | **2** |
| **Anti-inflammatory, anti-cancer [2]** | **Positive** | **595.1641** | **8.905** | **594.1568** | **** | **C_27_H_30_O_15_** | **Kaempferol4-glucoside 7-rhamnoside** | **3** |
| **Antioxidant, anti-inflammatory, anti-cancer** | **Positive** | **565.153** | **9.221** | **564.1456** | **** | **C_26_H_28_O_14_** | **Kaempferol3-rhamnoside7-xyloside** | **4** |
| **Anti-diabetic, anti-cancer [3]** | **Positive** | **415.1005** | **9.38** | **392.1113** | **** | **C_19_H_20_O_9_** | **Garcimangosone D** | **5** |
| **Anti-cancer**  **[34]** | **Positive** | **405.1992** | **10.241** | **382.2093** | **** | **C_24_H_30_O_4_** | **Assafoetidin** | **6** |
| **Antimicrobial, antioxidant, anti-inflammatory, anti-diabetic, and anti-cancer[4]** | **Positive** | **375.142** | **10.854** | **352.1529** | **** | **C_18_H_24_O_7_** | **Coriandrone D** | **7** |
| **Antioxidant, anti-inflammatory, anti-diabetic, and anti-cancer[5,6]** | **Positive** | **593.2694** | **12.186** | **570.2801** | **** | **C_32_H_42_O_9_** | **Ganoderic acid F** | **8** |
| **Anti-microbial [7]** | **Positive** | **357.1315** | **10.92** | **356.1244** | **** | **C_20_H_20_O_6_** | **Licoagrodione** | **9** |
| **Antimicrobial antioxidant, anti-inflammatory, and anti-cancer[8,9,10]** | **Positive** | **675.3841** | **14.284** | **674.3765** | **** | **C_37_H_50_N_6_O_6_** | **Hymenocardine** | **10** |
| **Antibacterial, antifungal[11]** | **Positive** | **473.288** | **18.802** | **472.2807** | **** | **C_28_H_40_O_6_** | **Macrocarpal B** | **11** |
| **Anti-cancer[12]** | **Positive** | **593.2742** | **25.287** | **592.2668** | **** | **C_35_H_36_N_4_O_5_** | **Pheophorbide A** | **12** |
| **Antioxidant, anti-inflammatory anti-diabetic, and anti-cancer[13]** | **Negative** | **153.019** | **1.651** | **154.0262** | **** | **C_7_H_6_O_4_** | **Gentisic acid** | **13** |
| **Antioxidant,anti-cancer[14]** | **Negative** | **1155.3196** | **9.052** | **1096.3054** | **** | **C_45_H_60_O_31_** | **Kaempferol 3-Osophorotrioside 7-Osophoroside** | **14** |
| **Antioxidant, anti-inflammatory,anti-cancer[15]** | **Negative** | **771.2327** | **7.811** | **726.2349** | **** | **C_33_H_42_O_18_** | **Naringin 6"-rhamnoside** | **15** |
| **Antioxidant, anti-inflammatory, anti-cancer[16]** | **Negative** | **593.1493** | **8.082** | **594.1577** | **** | **C_27_H_30_O_15_** | **Astragalin 7-rhamnoside** | **16** |
| **Antioxidant, anti-inflammatory, anti-cancer[17,44,45]** | **Negative** | **335.0773** | **8.731** | **290.0786** | **** | **C_15_H_14_O_6_** | **-Catechin(-)** | **17** |
| **Antioxidant, anti-inflammatory anti-diabetic, and anti-cancer[35]** | **Negative** | **577.155** | **9.027** | **578.1622** | **** | **C_27_H_30_O_14_** | **Lespenefril** | **18** |
| **Antioxidant, anti-inflammatory, anti-cancer[18]** | **Negative** | **133.0138** | **9.719** | **134.0206** | **** | **C_4_H_6_O_5_** | **L-Malic acid** | **19** |
| **Antioxidant ,anti-cancer [36,46]** | **Negative** | **285.0387** | **9.165** | **286.0461** | **** | **C_15_H_10_O_6_** | **Maritimetin** | **20** |
| **Antioxidant, anti-inflammatory anti-diabetic, and anti-cancer[19,20]** | **Negative** | **239.0546** | **9.862** | **180.0415** | **** | **C_9_H_8_O_4_** | **Caffeic acid** | **21** |
| **Anti-cancer[21].** | **Negative** | **405.2117** | **10.267** | **404..2036** | **** | **C_19_H_32_O_9_** | **Pisumionoside** | **22** |
| **Anti-inflammatory , anti-diabetic[22]** | **Negative** | **461.2027** | **10.276** | **462.2115** | **** | **C_21_H_34_O_11_** | **Patrinoside** | **23** |
| **Antioxidant, anti-inflammatory,and anti-cancer[23]** | **Negative** | **447.0917** | **9.165** | **448.099** | **** | **C_21_H_20_O_11_** | **8C- Galactosylluteolin** | **24** |
| **Antioxidant, anti-inflammatory[24]** | **Negative** | **723.3724** | **11.069** | **678.3738** | **** | **C_33_H_58_O_14_** | **Gingerglycolipid B** | **25** |
| **Antimicrobial antioxidant, anti-inflammatory, and anti-cancer[25]** | **Negative** | **807.4135** | **14.486** | **808.4205** | **** | **C_42_H_64_O_15_** | **Licoricesaponin B2** | **26** |
| **Antioxidant, anti-inflammatory[26]** | **Negative** | **327.2148** | **14.28** | **328.2221** |  | **C_18_H_32_O_5_** | **Corchorifatty acid F** | **27** |
| **Anti- inflammatory,and anti-cancer[27]** | **Negative** | **895.4661** | **14.283** | **896.4726** | **** | **C_46_H_72_O_17_** | **Momordin Ie** | **28** |
| **Antimicrobial antioxidant, anti-inflammatory, anti-diabetic, and anti-cancer[28]** | **Negative** | **1035.4896** | **14.081** | **1036.4945** | **** | **C_49_H_80_O_23_** | **Chinenoside I** | **29** |
| **Anti- cancer[29]** | **Negative** | **307.2242** | **24.403** | **308.2314** | **** | **C_19_H_32_O_3_** | **Obtusilactone A** | **30** |

In the positive ionization mode, several compounds were identified. Cis-zeatin-O-glucoside is a plant growth regulator that can reduce bud elongation .Norharman, another detected compound, has been previously reported to possess antioxidant and anti-cancer properties [1]. Additionally, several derivatives of kaempferol, such as Kaempferol-4'-glucoside-7-rhamnoside, were detected. Kaempferol itself is recognized for its anti-inflammatory, anti-cancer, and antioxidant effects [2]. Terpenoids like garcimangosone D and ganoderic acid F were also identified [3-6], both of which have been shown to exhibit anti-cancer, anti-diabetic, anti-inflammatory, and antioxidant properties. Other notable compounds detected included hymenocardine, 8C-galactosylluteolin, and chinenoside, which have been cited in the literature for their anti-cancer and anti-inflammatory properties [7-29].

In the negative ionization mode, phenolic compounds including gentisic acid, catechin, maritimetin, and caffeic acid were detected. These phenolic substances are recognized for their properties, which include anti-inflammatory, anti-diabetic, antioxidant, and anti-cancer effects.

Supplementary 3 provides summary of the various compounds detected in both positive and negative ionization modes, including several tentatively identified compounds with potential therapeutic relevance: Terpenoids such as Garcimangosone D, Ganoderic acid F, and Assafoetidin, which have been reported to exhibit anti-inflammatory, anti-diabetic, and anti-cancer properties; Flavonoids like Kaempferol-4'-glucoside-7-rhamnoside, Kaempferol-3-rhamnoside-7-xyloside, and Maritimetin, known for their antioxidant, anti-inflammatory, and anti-cancer activities; Phenolics such as Gentisic acid, Catechin, and Caffeic acid, which have been associated with anti-inflammatory, anti-diabetic, and anti-cancer effects; and the glycoside pisumionoside, which has demonstrated anti-cancer and anti-inflammatory properties.

The terpenoids, flavonoids, and phenolic compounds identified in the CGP extract have been extensively studied for their therapeutic potential. For instance, cucurbitane triterpenoids, such as Garcimangosone D and Ganoderic acid F, have been reported to possess potent anti-inflammatory, anti-diabetic, and anti-cancer properties. These compounds are believed to modulate key signaling pathways involved in the regulation of inflammatory mediators, glucose homeostasis, and cancer cell proliferation and survival.

Similarly, the flavonoids, including Kaempferol derivatives and Maritimetin, have demonstrated strong antioxidant activities, which can help mitigate oxidative stress-induced cellular damage. By preventing the activity of enzymes like cyclooxygenase (COX) and lipoxygenase (LOX), which are essential in the arachidonic acid cascade, these substances have also been demonstrated to have anti-inflammatory properties.

The phenolic compounds, such as Gentisic acid, Catechin, and Caffeic acid, have also been recognized for their ability to modulate various pathways related to inflammation, glucose metabolism, and cancer cell proliferation. These phytochemicals can potentially contribute to the observed anti-inflammatory, anti-diabetic, and anti-cancer activities of the CGP extract.

**References:**

**1.** Aaghaz S, Sharma K, Jain R, Kamal A. β-Carbolines as potential anticancer agents. Eur J Med Chem. 2021;216:113321. https://doi.org/10.1016/j.ejmech.2021.113321

**2.** Sururi AM, Tukiran T, Aisa ER, Raihan M. Identification of bioactive compounds and ADMET profile of stem bark of Syzygium samarangense and their potential as antibreast cancer and antiinflammatory. J Appl Pharm Sci. 2024;14(2):273-80. DOI: 10.7324/JAPS.2024.143017

**3.** Ahmed A. Evaluation of antidiabetic and anti-obesity potential and safety of a polyherbal remedy. Al-Azhar J Pharm Sci. 2022;65(1):229-45. DOI: 10.21608/AJPS.2022.223776

**4.** Marcucci MC, Oliveira CR, Spindola D, Antunes AA, Santana LY, Cavalaro V, et al. Molecular Dereplication and In Vitro and In Silico Pharmacological Evaluation of Coriandrum sativum against Neuroblastoma Cells. Molecules. 2022;27(17):5389. https://doi.org/10.3390/molecules27175389

**5.** Jiang J, Grieb B, Thyagarajan A, Sliva D. Ganoderic acids suppress growth and invasive behavior of breast cancer cells by modulating AP-1 and NF-κB signaling. Int J Mol Med. 2008;21(5):577-84.

**6.** Cho JY, Sadiq NB, Kim JC, Lee B, Hamayun M, Lee TS, et al. Optimization of antioxidant, anti-diabetic, and anti-inflammatory activities and ganoderic acid content of differentially dried Ganoderma lucidum using response surface methodology. Food Chem. 2021;335:127645. https://doi.org/10.1016/j.foodchem.2020.127645

**7.** Li W, Asada Y, Yoshikawa T. Antimicrobial flavonoids from Glycyrrhiza glabra hairy root cultures. Planta Med. 1998;64(08):746-7. DOI: 10.1055/s-2006-957571.

**8.** Sofidiya MO, Odukoya OA, Adedapo AA, Mbagwu HOC, Afolayan AJ, Familoni OB. Investigation of the anti-inflammatory and antinociceptive activities of Hymenocardia acida Tul.(Hymenocardiaceae). Afr J Biotechnol. 2010;9(49):8454-9.

**9.** Amom TT, Yahwe SR, Vershima AJ. Phytochemical and medicinal activities of Hymenocardia acida Tul (Euphorbiaceae): A Review. J Nat Prod Plant Resour. 2013;3:11-6.

**10.** Adeleke GE, Owolabi OQ, Berena GA, Ajani RA, Adeyi RO, Orisadiran PK, et al. Chemical characterization of Hymenocardia acida stem bark extract and modulation of selected enzymes in Kidney and Heart of Wistar rats. Med Res Arch. 2023;11(9). https://doi.org/10.18103/mra.v11i9.4288

**11.** Yamakoshi Y, Murata M, Shimizu A, Homma S. Isolation and Characterization of Macrocarpals B—G Antibacterial Compounds from Eucalyptus macrocarpa. Biosci Biotechnol Biochem. 1992;56(10):1570-6. https://doi.org/10.1271/bbb.56.1570

**12.** Hajri A, Wack S, Meyer C, Smith MK, Leberquier C, Kedinger M, Aprahamian M. In vitro and in vivo efficacy of photofrin and pheophorbide a, a bacteriochlorin, in photodynamic therapy of colonic cancer cells. Photochem Photobiol. 2002;75(2):140–148. https://doi.org/10.1562/0031-8655(2002)0750140IVAIVE2.0.CO2

**13.** Fuentes-Retamal S, Sandoval-Acuña C, Peredo-Silva L, Guzmán-Rivera D, Pavani M, Torrealba N, et al. Complex mitochondrial dysfunction induced by TPP+-Gentisic acid and mitochondrial translation inhibition by doxycycline evokes synergistic lethality in breast cancer cells. Cells. 2020;9(2):407. DOI: 10.3390/cells9020407

**14.** Vrchovská V, Sousa C, Valentão P, Ferreres F, Pereira JA, Seabra RM, Andrade PB. Antioxidative properties of tronchuda cabbage (Brassica oleracea L. var. costata DC) external leaves against DPPH, superoxide radical, hydroxyl radical and hypochlorous acid. Food Chem. 2006;98(3):416-25. https://doi.org/10.1016/j.foodchem.2005.06.019

**15.** Wang Y, Li X, Lv H, Sun L, Liu B, Zhang X, Xu X. Therapeutic potential of naringin in improving the survival rate of skin flap: A review. Front Pharmacol. 2023;14:1128147. DOI: 10.3389/fphar.2023.1128147

**16.** Kaidash OA, Kostikova VA, Udut EV, Shaykin VV, Kashapov DR. Extracts of Spiraea hypericifolia L. and Spiraea crenata L.: The Phenolic Profile and Biological Activities. Plants. 2022;11:2728. https://doi.org/10.3390/plants11202728

**17.** FeiYan F, Sang LiXuan SL, Jiang Min JM. Catechins and their therapeutic benefits to inflammatory bowel disease. Molecules. 2017. DOI:10.3390/molecules22030484

**18.** Arslan ME. Anticarcinogenic properties of malic acid on glioblastoma cell line through necrotic cell death mechanism. MANAS J Eng. 2021;9(1):22-9. https://doi.org/10.51354/mjen.848282

**19.** Chiang EP, Tsai SY, Kuo YH, Pai MH, Chiu HL, Rodriguez RL, Tang FY. Caffeic acid derivatives inhibit the growth of colon cancer: involvement of the PI3-K/Akt and AMPK signaling pathways. PLoS One. 2014;9(6):e99631. https://doi.org/10.1371/journal.pone.0099631

**20.** Matowane GR, Ramorobi LM, Mashele SS, Bonnet SL, Noreljaleel AEM, Swain SS, Makhafola TJ, Chukwuma CI. Novel Caffeic Acid - Zinc Acetate Complex: Studies on Promising Antidiabetic and Antioxidative Synergism Through Complexation. Med Chem. 2023;19(2):147–162.

https://doi.org/10.2174/1573406418666220620144601

**21.** Kuang H, Xia Y, Yang B, Wang Q, Lü S. Sesquiterpene Glucosides from Chloranthus japonicus Sieb. Chem Biodivers. 2008;5(9):1736–1742. https://doi.org/10.1002/cbdv.200890162

**22.** Liu Z, Wang M, Liu Y, Ren M, Xi X, Li S, Kang W. Patrinoside and Patrinoside A from Patrinia scabiosaefolia Improve Insulin Resistance by Inhibiting NF-κB, MAPK Pathways and Oxidative Stress in RAW264.7 and 3 T3-L1 Cells. Oxid Med Cell Longev. 2023;2023:9069645. https://doi.org/10.1155/2023/9069645

**23.** Khalil HE, Ibrahim HIM, Ahmed EA, Emeka PM, Alhaider IA. Orientin, a bio-flavonoid from Trigonella hamosa L., regulates COX-2/PGE-2 in A549 cell lines via miR-26b and miR-146a. Pharmaceuticals. 2022;15(2):154.

https://doi.org/10.3390/ph15020154

**24.** Kiem PV, Minh CV, Nhiem NX, Cuong NX, Tai BH, Quang TH, Anh HleT, Yen PH, Ban NK, Kim SH, Xin M, Cha JY, Lee YM, Kim YH. Inhibitory effect on TNF-α-induced IL-8 secretion in HT-29 cell line by glyceroglycolipids from the leaves of Ficus microcarpa. Arch Pharm Res. 2012;35(12):2135–2142.

https://doi.org/10.1007/s12272-012-1210-8

**25.** Li F, Liu B, Li T, Wu Q, Xu Z, Gu Y, et al. Review of constituents and biological activities of triterpene saponins from Glycyrrhizae Radix et Rhizoma and its solubilization characteristics. Molecules. 2020;25(17):3904.

https://doi.org/10.3390/molecules25173904

**26.** Wu P, Ben T, Zou H, Chen Y. PARAFAC modeling of dandelion phenolic compound fluorescence relation to antioxidant properties. J Food Meas Charact. 2022;16(4):2811-9. https://doi.org/10.1007/s11694-022-01389

**27.** Kwon H, Park S, Lee S, Lee DK, Yang CH. Determination of binding constant of transcription factor AP-1 and DNA. Application of inhibitors. Eur J Biochem. 2001;268(3):565–572. https://doi.org/10.1046/j.1432-1327.2001.01897.x

**28.** Wang H, Zheng Q, Dong A, Wang J, Si J. Chemical Constituents, Biological Activities, and Proposed Biosynthetic Pathways of Steroidal Saponins from Healthy Nutritious Vegetable—Allium. Nutrients. 2023;15(9):2233.

https://doi.org/10.3390/nu15092233

**29.** Wang HM, Cheng KC, Lin CJ, Hsu SW, Fang WC, Hsu TF, Chiu CC, Chang HW, Hsu CH, Lee AY. Obtusilactone A and (-)-sesamin induce apoptosis in human lung cancer cells by inhibiting mitochondrial Lon protease and activating DNA damage checkpoints. Cancer Sci. 2010;101(12):2612–2620. https://doi.org/10.1111/j.1349-7006.2010.01701.x
